# Integrative whole-genome sequence analysis reveals roles of regulatory mutations in *BCL6* and *BCL2* in follicular lymphoma

**DOI:** 10.1038/s41598-017-07226-4

**Published:** 2017-08-01

**Authors:** Kirill Batmanov, Wei Wang, Magnar Bjørås, Jan Delabie, Junbai Wang

**Affiliations:** 1 Department of Pathology, Oslo University Hospital – Norwegian Radium Hospital, Montebello, 0310 Oslo Norway; 2 Institute for Cancer Research and Molecular Medicine, Norwegian University of Science and Technology, Trondheim, Norway; 3 Laboratory Medicine Program, University Health Network and University of Toronto, Toronto, Ontario Canada; 4 Department of Microbiology, Oslo University Hospital and University of Oslo – 0027, Oslo, Norway

## Abstract

The contribution of mutations in regulatory regions to tumorigenesis has been the subject of many recent studies. We propose a new framework for integrative analysis of genome-wide sequencing data by considering diverse genetic information. This approach is applied to study follicular lymphoma (FL), a disease for which little is known about the contribution of regulatory gene mutations. Results from a test FL cohort revealed three novel highly recurrent regulatory mutation blocks near important genes implicated in FL, *BCL6* and *BCL2*. Similar findings were detected in a validation FL cohort. We also found transcription factors (TF) whose binding may be disturbed by these mutations in FL: disruption of FOX TF family near the *BCL6* promoter may result in reduced *BCL6* expression, which then increases *BCL2* expression over that caused by *BCL2* gene translocation. Knockdown experiments of two TF hits (*FOXD2* or *FOXD3*) were performed in human B lymphocytes verifying that they modulate *BCL6*/*BCL2* according to the computationally predicted effects of the SNVs on TF binding. Overall, our proposed integrative analysis facilitates non-coding driver identification and the new findings may enhance the understanding of FL.

## Introduction

High-throughput sequencing techniques have allowed researchers to study cancer-associated genetic and epigenetic alterations in great detail. International consortia such as The Cancer Genome Atlas (TCGA) and the International Cancer Genome Consortium (ICGC) have sequenced thousands of samples of tumor and normal tissues, making it possible to survey the overall picture of molecular aberrations in cancer^1, 2^, as well as to investigate particular mechanisms of oncogenesis^3, 4^. Since the cost of genome-wide sequencing experiments reduced significantly in recent years, analysis of non-coding DNA variations has become an intensively researched area^5^, and several computational methods have been developed for this purpose. These include **CADD** ^6^ and **FunSeq**.**2** ^7^ that integrate various data sources (e.g. conservation scores, predicted transcription factor (TF) binding sites, chromatin state marks, measured ChIP-Seq peaks of TF binding, and protein-protein interactions) to detect functional variants in non-coding regions. Other tools (e.g. **is-rSNP** ^8^, **sTRAP** ^9^) are based on the hypothesis that non-coding variants affect gene expression primarily by changing protein-DNA interaction^10^. In these methods, TF-DNA binding affinity changes due to a given single nucleotide variant (SNV) are estimated by scanning a DNA sequence with known TF position weight matrices (PWMs), through either a statistical method or a machine learning technique. Other methods explore alternatives to PWM as the binding model, for example DeepSEA^11^, which uses a neural network to estimate binding affinity changes for a limited set of TFs. Recently, we developed a biophysical model, BayesPI-BAR^12^, to estimate the significance of TF-DNA binding affinity changes caused by a small non-coding variant, which not only includes PWMs as TF-DNA affinity models, but also considers characteristics of direct binding sites vs. indirect ones in *in vivo* TF-DNA interaction, as well as variable chemical potential^13^. BayesPI-BAR has provided the best prediction accuracy among the pure sequence based tools (is-rSNP and sTRAP). It also shows better performance compared to aforementioned integration methods (CADD and FunSeq. 2) when distinguishing functional regulatory variants from random ones in human genome^12^.

Although several programs have been designed for identifying functional regulatory mutations, and were already applied on a large number of diverse cancer genomes from ICGC and TCGA^6, 7, 14^, the usefulness of *in silico* methods for detection of unknown functional regulatory mutations in a specific cancer by using whole-genome sequencing data is not fully explored. In this work, we focus on follicular lymphoma (FL), which is a common indolent non-Hodgkin lymphoma. It is an incurable but clinically indolent malignancy with average 5-year survival rate of 0.74^15, 16^. FL patients often undergo a series of remissions and relapses, and ultimately, the disease may transform into diffuse large B cell lymphoma (DLBCL)^17^. Multiple studies have investigated the genetic basis of FL. Somatic mutations in genes coding for chromatin-modifying enzymes such as KTM2D, CREBBP, EP300, EZH2, HIST1H1E^4, 17–21^ in addition to the chromosomal translocation t(14; 18) likely constitute early events^17^. The result of t(14;18) translocation is the constitutive expression of *BCL2*, an anti-apoptotic protein. This leads to a survival advantage for the cells during proliferation in the germinal center, because *BCL2* is not normally expressed at this stage of B cell differentiation. The *BCL2* expression is controlled by the *IgH* enhancer, but its over-expression alone is insufficient to cause FL^22^. In addition, the t(14; 18) mutation is often found in healthy individuals^23^. Thus, other genetic events contribute to FL pathogenesis.

FL is a relatively homogenous lymphoma subtype^24^, which is suited for studying the genetic basis of disease and finding possible therapies to target the key genetic alternation. Recently, several studies^17, 24^ performed whole-genome sequencing on FL, but results are mainly focused on somatic mutations within the genes. This may be caused by a lack of proper tools to interpret the large number of non-coding variations in FL. Gene expression and function is affected not only by mutations in the genes, but also by non-coding mutations in regulatory regions^10, 25^, thus it is essential to investigate the relationship between the non-coding mutation and FL. Motivated by aforementioned challenges, we developed a novel genome-wide analysis pipeline based on the newly updated BayesPI-BAR program, and applied it on a set whole-genome sequencing datasets of FL patients^26^, to explore unknown regulatory mutation in FL in the present study.

In this work, we propose a new integrated approach, which combines diverse information (the spatial distribution of SNVs, the differential gene expression profiles between tumor and normal samples, and the biophysical modeling of TD-DNA binding affinity changes that are caused by either a given SNV or patient-specific SNVs), to find putative functional non-coding sequence variations across multiple FL cancer genomes. Based on the proposed new method, we discovered several novel mutation blocks near the promoter regions of *BCL2* and *BCL6* genes. We relate the presence of these mutations to the binding of several TFs in the promoter regions and analyze the effect on gene expression. Our findings add another layer to the complex pathogenesis of FL.

## Results

### Selection of reliably called SNVs

Genome-wide sequencing data of 14 tumor-normal paired FL patients were obtained from International Cancer Genome Consortium (ICGC)^26^. We aligned all sequencing data of 14 tumor-normal paired samples to GRCh37 human genome^27^, and used both Strelka^28^ and MuTect^29^ to call SNVs. Six of these fourteen patients were studied in an earlier paper^2^, and genome-wide mutations, detected by SAMtools^30^, were made publicly available^24^. The overlap between the published genome-wide mutations of 6 patients and our called ones from Strelka is ~95%, indicating a good quality of mutation calls. Since running MuTect on the genome-wide sequencing data is very time consuming, we only called mutations located within ±10000 bp to the transcription start sites (TSS). In these regions, the overlap of called mutations between the MuTect and Strelka is ~66%. An intersection of mutations calls from both programs was used for the downstream data analysis. The reason is that mutations identified by two programs are more reliable than those called by a single one. The same approach to mutation calling was used in a previous study of functional cancer regulatory mutations^14^. Thus, these reliably called SNVs from two programs for 14 tumor-normal paired FL patients will be used as a test cohort in further investigation.

### A new integrated method to find functional regulatory mutations

**Figure 1:**
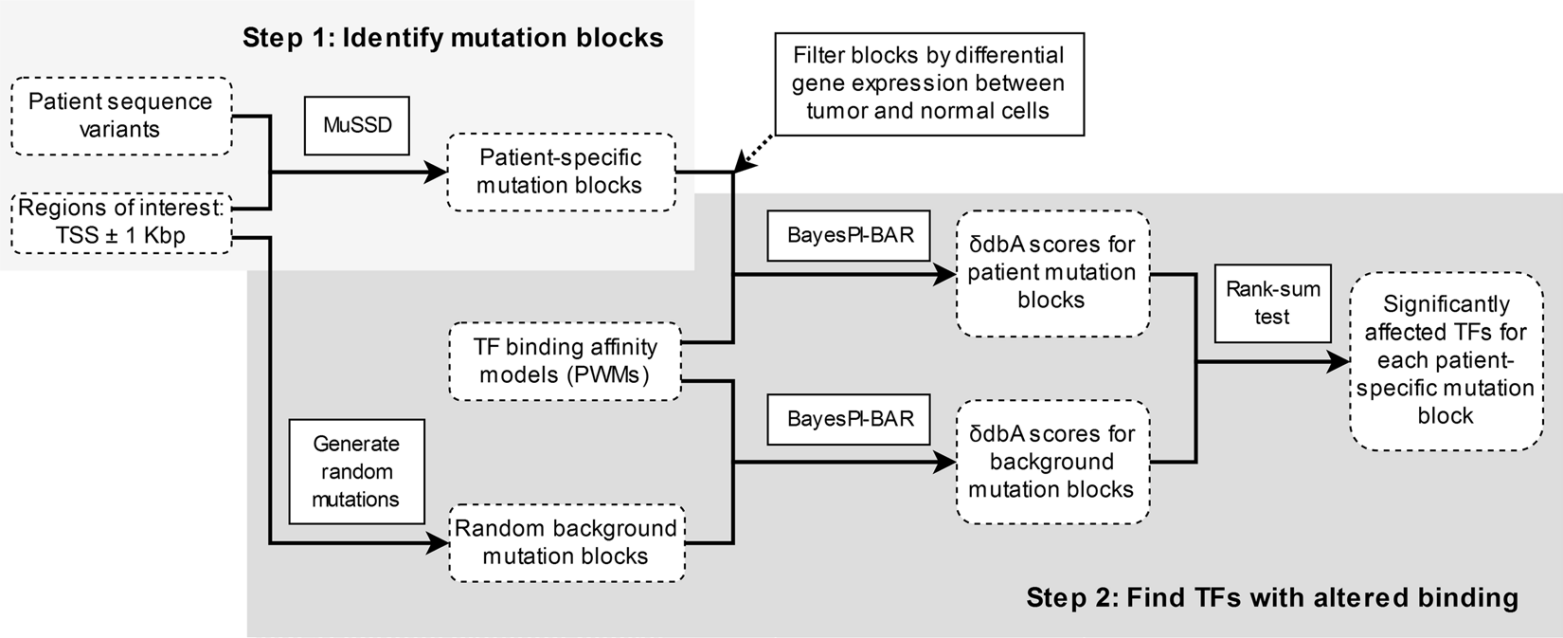
Flowchart of the BayesPI-BAR2 pipeline. The pipeline inputs are: the sequence variants for all patients, the definition of the regions of interest (TSS ± 1 Kbp), and the set of PWMs of known TFs. In Step 1, the patient-specific mutation blocks are identified using the MuSSD algorithm. In Step 2, the TF binding affinity changes in these blocks are predicted by BayesPI-BAR, and compared to that of random background mutations, to obtain a list of significantly affected TFs at each block.

Previously developed tools for non-coding somatic mutation analysis treat each variant individually. In this work, we develop a new method, based on the original BayesPI-BAR algorithm, to evaluate possible TF binding effects by considering all patient mutations simultaneously. The flowchart of the new integrated method - BayesPI-BAR2 - is outlined in Fig. 1, which consists of two main steps as explained below. First, we identify non-coding DNA regions that are highly mutated in the patient samples (*mutation blocks*), using Mutation filtering based on the Space and Sample Distribution – MuSSD – algorithm. This algorithm finds regions which have high density of mutations in multiple patients (see Materials and Methods for detailed description). Then, we use the original BayesPI-BAR program to compute the DNA binding affinity change (δdbA) for each TF in each patient-specific mutation block. For the same TF, a set of δdbA from patients are compared to a set of δdbA obtained from randomly generated background mutations, by the Wilcoxon rank-sum test. In this way, we are able to identify functional regulatory mutation blocks that significantly affect the TF binding.

### First step BayesPI-BAR2 analysis: Identification of functional regulatory mutation blocks and the target genes in FL

Usually, a TF binding motif appearing at a gene promoter region indicates that the TF may regulate the gene activity^31^. By searching for somatic SNVs that locate in these regions (e.g., ±1000 bp to TSS) and disrupt the putative TF binding motifs, we may identify functional non-coding mutations in cancer. For that reason, in the first step of BayesPI-BAR2 analysis, we only consider mutations at the promoter regions (±1000 bp to TSS) of all available protein-coding genes in the GENCODE^32^. There are 795 reliably called SNVs in these regions for 14 FL patients. To further reduce the number of SNVs for which we need to evaluate the potential impact on TF binding affinity changes, the 795 SNVs were investigated by MuSSD algorithm. After grouping these reliably called mutations into regulatory mutation blocks based on their space and sample frequency distributions, the number of interesting SNVs for FL is dropped to 147.

**Figure 2:**
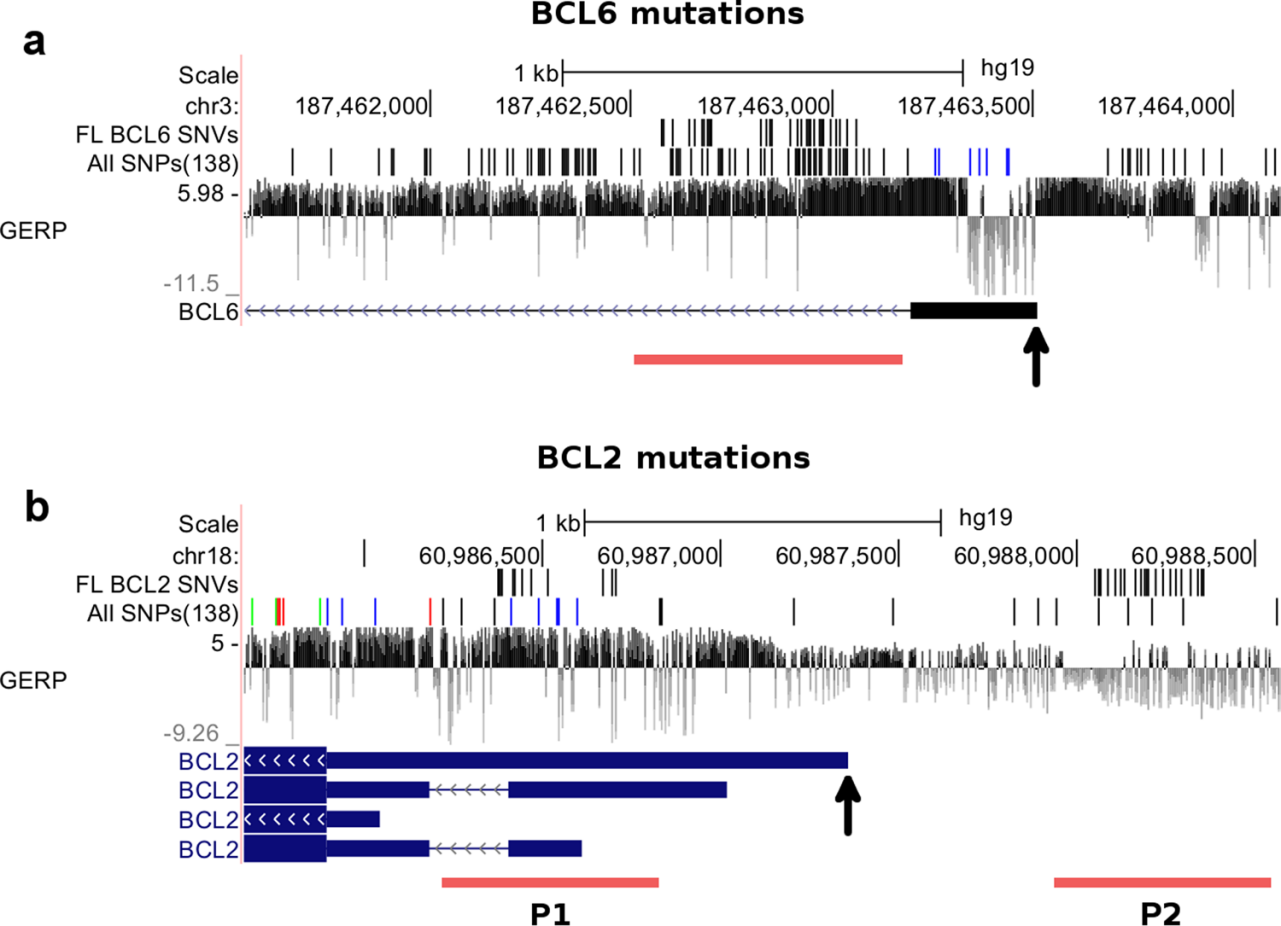
Predicted regulatory mutation blocks in *BCL6* and *BCL2* from a test cohort of 14 FL patients. Genome Browser overview of *BCL6* and *BCL2* mutation blocks. For reference, all mutations from dbSNP and GERP conservation scores are given. The reference genome annotations are displayed below, with solid bars representing exons and thin lines representing introns. Arrows on introns indicate direction of transcription. Black arrow marks the TSS. Red bars represent locations of the predicted regulatory mutation blocks of *BCL6* and *BCL2*. **P1** and **P2** represent *BCL2* regulatory mutation blocks one and two, respectively.

**Figure 3:**
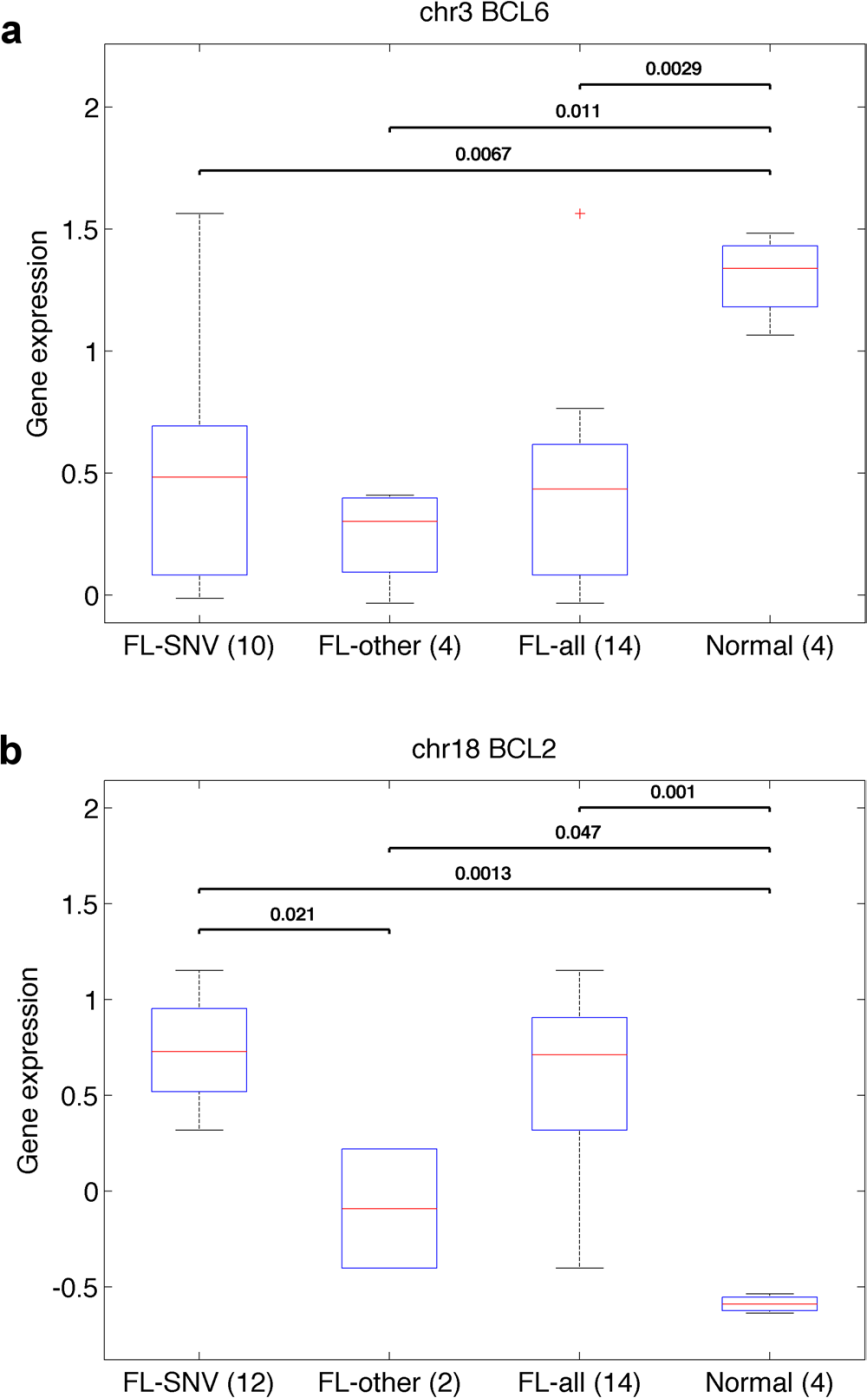
Box plot of gene expression levels of FL and GCB control samples. Here, FL-SNV represents FL patients with regulatory mutation blocks, FL-other represents FL patients without the regulatory mutation blocks, FL-all mean all 14 FL patients of test cohort, Normal means GCB control samples. The number of FL patients/control samples in the category is given in parentheses. P-value of the significance of KS test for difference between gene expressions in two categories is given above the bar connecting corresponding categories.

These identified mutation blocks (Fig. 2 and Supplementary Figure 1) are located near 8 genes: *BCL6*, *HIST1H2BM*, *LTB*, *BIRC3*, *TCL1A*, *IL4R*, *BCL2*, and *IGLL5*. The expression levels of these 8 genes were compared between the 14 FL patients and 4 GCB control samples by using a two-sample Kolmogorov-Smirnov goodness-of-fit hypothesis test (KS-test)^33^. With a P-value threshold at 0.05, only 3 genes (*BCL6*, *BCL2*, and *HIST1H2BM*), containing the regulatory mutation blocks, show significant differential expression between the tumor and normal samples (Fig. 3 and Supplementary Figure 2). This has been further confirmed by the baySeq program^34^ (Supplementary Table 1). The regulatory mutation blocks of these three genes contain 34, 40, and 2 SNVs near TSS of *BCL6*, *BCL2*, and *HIST1H2BM*, respectively. These SNVs are spanned to 10, 12, and 2 FL patients for *BCL6*, *BCL2*, and *HIST1H2BM*, respectively. At least one called SNV is present in the regulatory mutation blocks of either *BCL6* or *BCL2* in each FL sample, indicating the importance of the mutations for FL pathogenesis. Thus, three putative functional regulatory mutation blocks near *BCL6* and *BCL2* (Fig. 2) were chosen for further analysis in the second step of the BayesPI-BAR2 pipeline (Fig. 1). The reason is that the 74 SNVs from the three selected mutation blocks are not only distributed among all 14 FL patients, but also associated with differentially expressed genes between the tumor and normal samples. The new analysis may reveal a relationship between these somatic non-coding mutations and gene regulation in FL.

Additionally, we computed the nucleotide change statistics for these SNVs (Supplementary Table 2a). The distribution of nucleotide changes resembles the signature 1B as defined by Alexandrov *et al*.^2^, the most common mutation signature, which is associated with old age. This is expected as FL occurs at median age of 60 years. Aberrant somatic hypermutation (aSHM) was previously observed in FL, at least near *BCL6* ^35^. We have tested mutations in the three regulatory mutation blocks near *BCL6* and *BCL2* for SHM hotspot motifs (DGYW/WRCH; Supplementary Methods). Although the sample size is small, we could establish the influence of aSHM in the two blocks near *BCL2* (P < 0.03 for two-sided Binomial test), but not the *BCL6* block (P = 0.47; Supplementary Table 2b). Finally, there is a difference (Fig. 3) between the regulatory mutation blocks of *BCL6* and those of *BCL2* with regard to influence on gene activity. The differential expression of *BCL6* is significant between the FL patients and the control samples, for both with (10 FL; P < 0.007 for two-sample KS-test) and without (4 FL; P < 0.012) the regulatory mutation blocks. However, differential expression of *BCL2* is much more pronounced for patients with the regulatory mutation blocks (12 FL; P < 0.0013) than those without the regulatory mutation blocks (2FL; P < 0.05).

### Verification of updated BayesPI-BAR on 67 known human regulatory SNVs

In the new BayesPI-BAR program, several improvements to the computation of shifted differential binding affinity (δdbA) scores^12^ are made, the total computation times is reduced ~70% by applying an early stopping rule on the random background sampling, and the possibility to distribute jobs across several computer nodes was added. In original BayesPI-BAR publication^12^, ~70% of tested SNVs have true TFs (TF-DNA interactions that are known to be affected by the SNVs) ranked in the top 10 predictions. We verified the new BayesPI-BAR program on the same 67 known human regulatory SNVs, and found that ~74% of 67 SNVs have true TFs ranked among the top 10 predicted TFs, using an expected P-value for direct TF binding <0.1 and the magnitude of δdbA at the top 20% (Supplementary Figure 3). The additional filtering by δdbA discards about 10% of irrelevant TFs from the rankings without affecting the accuracy. With the same parameters, ~80% of known SNVs have true TFs ranked in the top 15. However, the results are not improved when we considered the top 20 ranked predictions. Therefore, in the subsequent data analysis, aforementioned BayesPI-BAR parameters and top 15 predictions were considered by the new BayesPI-BAR program, to predict TFs that are associated with the putative functional regulatory mutation blocks in FL.

### Individual SNV analysis: *BCL6* regulatory mutation block

As a first step toward the analysis of detected putative functional regulatory mutations, we simply applied the new BayesPI-BAR on each individual SNV. The output of BayesPI-BAR is a list of TFs whose binding is potentially affected by a given SNV, ranked by the predicted effect size. These rankings were then visualized and examined to identify any TFs that appear to be affected frequently across patients, which may indicate that their regulation tends to be disrupted by the mutations. By applying the updated version of BayesPI-BAR on the 61 bp long DNA sequences centered at each individual SNV, we analyzed all SNVs that are located in the functional regulatory mutation block of *BCL6* (34 putative functional non-coding SNVs). We selected the top 15 TFs, the binding of which was positively and negatively affected by each SNV, after scanning 2065 human TF PWMs for 34 SNVs in the *BCL6* regulatory mutation block (~500 bp in Fig. 2). The top 35 TFs affected positively and negatively with regard to binding affinity are shown in Supplementary Figure 4a and b. The ~25 to ~35% of predicted TFs with very low expression, according to RNA-Seq data, were filtered out (Supplementary Table 3). FOXL1, TBP, TCF4, BPTF (FAC1), TEAD2 (ETF), CEBPB, and YY1 are the TFs with positive binding affinity change by the mutations (e.g. the binding affinity of FOXL1 is the most frequently changed one by SNVs; 8 out of 34 SNVs). Three of these TFs (CEBPB, BPTF and YY1) are associated with negative regulation of transcription from RNA polymerase II promoter (Gene Ontology study by DAVID on-line tool). This may explain why *BCL6* gene is downregulated in FL compared to control germinal center cells (Fig. 3). CEBPB, TBP, FOXL1, NFATC2, FOXO1, POU2F1, FOXJ2, GTF2I (TFIII), GMEB2, ETS1, and BRCA1 are the TFs of which mutations negatively influence binding. CEBPB is the most often modified by SNVs; 10 out of 34 SNVs. CEBPB, FOXL1, POU2F1, FOXO1, BRCA1 are associated with regulation of RNA metabolism whereas TBP and NFATC2 are related to transcription factor activity and RNA polymerase II core promoter proximal region sequence-specific DNA binding. Thus, the loss of TBP and NFACT2 binding, at the promoter of *BCL6*, may result in inactivation of *BCL6* in the FL patients. Additionally, some of the positively affected TFs (i.e. BPTF, CEBPB, TCF4, and YY1) are linked to chromatin remodeling and enhancer binding, which indicate the chromatin and enhancer activities may also be responsible for the *BCL6* differential expression between the FL and normal control samples.

### Individual SNV analysis: *BCL2* regulatory mutation blocks

Using the same analysis as for *BCL6* in the previous section, we investigated disrupted TF-DNA interactions in *BCL2* regulatory mutation blocks (40 putative functional non-coding SNVs). In Supplementary Figure 5a, we display the heat-map of the 35 most frequent TFs with increased binding affinities at *BCL2* promoter. STAT6, ARNT, ZEB1, STAT5A, AHR, CEBPB, STAT1, STAT3, MZF1, and TBP are the top TFs in the *BCL2* regulatory mutation blocks. STAT5A and STAT3 are linked to anti-apoptosis, and CEBPB and ZEB1 are related to enhancer binding and chromatin binding/remodeling. In Supplementary Figure 5b, we list the top 35 TFs with decreased binding affinities at *BCL2* promoter, with ZEB1, CEBPB, AP4 (TFAP4), STAT1, MYC, and STAT6 being the most frequently affected TFs (e.g. ZEB1 is the top modified one – 10 out of 40 SNVs affect its binding affinity). MYC is linked to apoptosis and lymphoma whereas ZEB1 and CEBPB are enhancer binding proteins. Since the expression level of *BCL2* is significantly higher in FL patients than in normal control samples (Fig. 3), the regulatory mutation blocks (e.g. 40 SNVs spread at two major blocks; Fig. 2) of *BCL2* may directly contribute to increased *BCL2* expression. These results suggest that the high *BCL2* expression in FL (Fig. 3) may at least partly be related to the deregulation of important TFs at the regulatory mutation blocks of *BCL2*.

### Relationship between the functional regulatory mutation blocks and the super-enhancer

The application of the new BayesPI-BAR on mutated sequences with individual SNV indicates that the regulatory mutation blocks of both *BCL2* and *BCL6* may influence the enhancer binding protein and chromatin activity. Therefore, we searched for overlapping regions between the promoter region (i.e. ±1000 bp to TSS of either *BCL2* or *BCL6*) and the known super-enhancers in human genome by using dbSUPER^36^. There are two super-enhancers overlapping with the promoter region of *BCL2*. The two super-enhancers were detected in DHL6 and Toledo (human diffuse large B cell lymphomas cell) cell lines, respectively. For *BCL6*, we did not find any known super-enhancers that are overlapping with its promoter region. For that reason, we only tested how many TFs may bind at the promoter region of *BCL2* according to all available *in vivo* experiments in the literature by using ReMap^37^. There are almost 56 TFs bound at the promoter (±1000 bp to TSS) of *BCL2* in various cell lines and conditions. Some of TFs are predicted by the new BayesPI-BAR to be affected by mutations in the current study, among which the top four TFs, MYC, ZEB1, CEBPB, and STAT1, which have decreased binding affinities at the promoter of *BCL2* (Supplementary Figure 5b). Other bound TFs, not in the prediction list, are related to long distance DNA interactions (i.e. YY1 and CTCF), and histone modifications (i.e. BRD2 and BRD4). Therefore, the long distance chromosome-chromosome interaction, chromatin and enhancer binding, and the epigenetic modifications may all be involved in FL^38, 39^.

### Second step BayesPI-BAR2 analysis: *BCL6* regulatory mutation block

By analyzing individual SNVs (a DNA sequence containing only one nucleotide variation) with the BayesPI-BAR, we did not find a TF that is affected by more than 30% of SNVs that come from the same regulatory mutation block. The simple analysis of individual SNV has three disadvantages: 1) it ignores the fact that several nearby mutations from the same patient may contribute to TF binding affinity changes cooperatively, 2) ranking of TFs affected by sequence variations does not consider the magnitude of TF binding affinity changes (e.g., a top ranked TF may have a very small δdbA value), and 3) the importance of affected TFs is based on a manual examination of the rankings with unclear criteria. These drawbacks motivated us to develop the second step of BayesPI-BAR2 pipeline (Fig. 1) to improve the prediction, where we first designed patient-specific alternative sequences (regulatory mutation blocks that contains all nucleotide variations from the same patient) to investigate how many patients may be affected by a TF’s affinity change through SNVs located in a predefined regulatory mutation block (e.g., ~350 bp and ~500 bp in *BCL2* and *BCL6*, respectively; Fig. 2). Then, we used Wilcoxon rank-sum test to compare the δdbA values of a patient group from a top ranked TF with δdbA of randomly generated mutation blocks, and report the TFs which are significantly affected at P < 0.05 level (Bonferroni-corrected). For a more detailed description, please refer to the Methods section.

**Figure 4:**
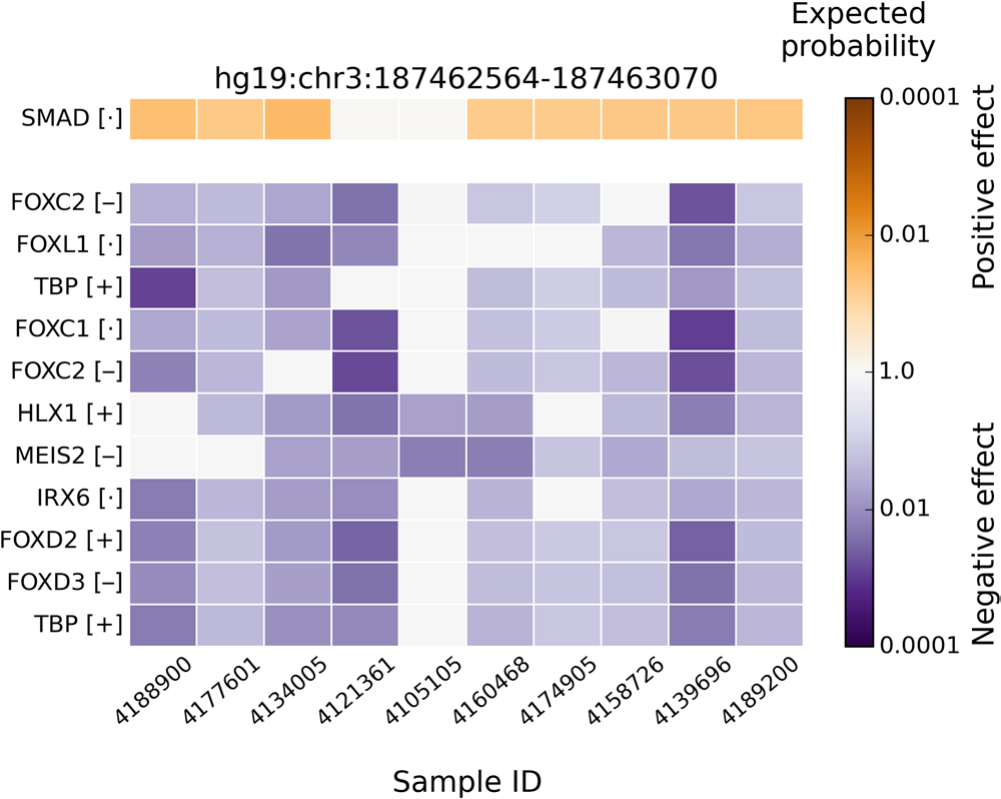
TFs significantly affected at *BCL6* patient-specific regulatory mutation block. The row labels are the TF names, and the column labels are patients with the regulatory mutation block near the *BCL6* TSS. TF names are repeating when several alternative PWMs for a single TF are significantly affected. The color encodes the expected probability that the TF will be affected by random mutations as strongly as by the patient mutations, on the logarithmic scale. The positive and negative affinity changes are colored orange and blue, respectively. In square brackets inside row labels: “+” means that the TF is highly expressed in patient samples (top 25%), “•” means average expression, “−” means low expression (lower 25%). TFs with very low expression (RPKM < 0.03) were filtered out. Only TFs with significant changes (P < 0.05 after Bonferroni correction) across all patients are shown.

Ranking results of the top 35 most affected TFs at the patient-specific regulatory mutation blocks of *BCL6* are shown by the heat-maps in Supplementary Figure 6a and Supplementary Figure 6b for positive and negative changes, respectively. Figure 4 shows TFs with significant binding affinity changes in BCL6 regulatory mutation block based on the Wilcoxon rank-sum test (P < 0.05 after Bonferroni correction). The color represents the expected probability of an observed δdbA value to occur in the background δdbA distribution. Supplementary Table 4 contains more detailed information of significantly affected TFs. For the positive change of TF binding affinities, only SMAD family passed significance test, where the binding of SMAD is significantly affected by sequence variations in 8 out of 10 FL patients. However, for the negative modifications of TF binding affinities, there are nine TFs (TBP, five members of the FOX family, IRX6, MEIS2, and HLX1) that are predicted to be significantly affected by sequence variants in *BCL6* regulatory mutation block. Both TBP and FOX proteins’ binding affinities are significantly decreased in 9 out of 10 FL patients. Most of these negatively affected TFs (e.g. TBP, FOXL1, and FOXD2) are playing a key role in the activation of eukaryotic genes transcribed by RNA polymerase II. Thus, the reduced *BCL6* gene expression in FL with respect to normal germinal center cells (Fig. 3a) may be caused by the loss of TBP or FOX protein binding in the regulatory mutation blocks.

### Second step BayesPI-BAR2 analysis: *BCL2* regulatory mutation blocks

**Figure 5:**
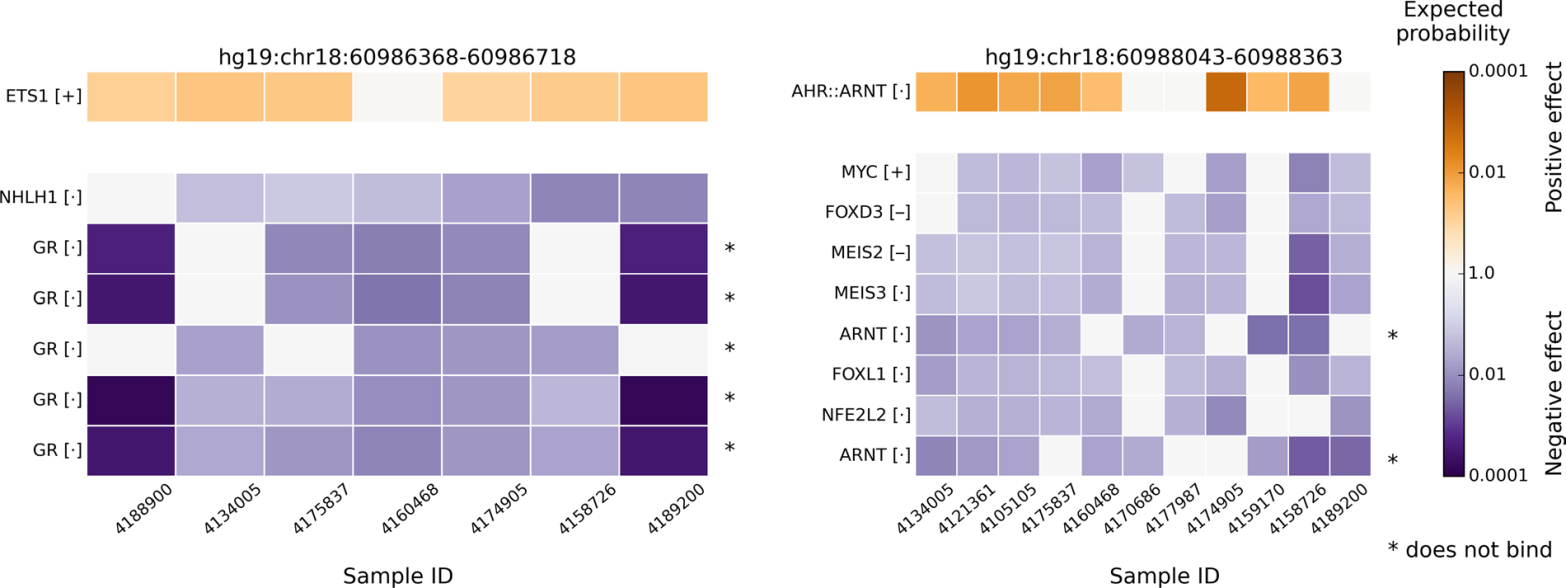
TFs significantly affected at *BCL2* patient-specific regulatory mutation blocks. The row labels are the TF names, and the column labels are patients with a regulatory mutation block near the *BCL2* TSS: block one (left) and block two (right). TF names are repeating when several alternative PWMs for a single TF are significantly affected. The color encodes the expected probability that the TF will be affected by random mutations as strongly as by the patient mutations, on the logarithmic scale. The positive and negative affinity changes are colored orange and blue, respectively. In square brackets inside row labels: “+” means that the TF is highly expressed in patient samples (top 25%), “•” means average expression, “−” means low expression (lower 25%). TFs with very low expression (RPKM < 0.03) were filtered out. Only TFs with significant changes (P < 0.05 after Bonferroni correction) across all patients are shown.

The predicted TFs binding affinity changes in *BCL2* patient-specific regulatory mutation blocks are shown by Supplementary Figure 7a and Supplementary Figure 7b for the positive and the negative modifications, respectively. Figure 5 shows TFs with significant binding affinity changes based on the Wilcoxon rank-sum test (Bonferroni corrected P < 0.05). For more detailed results please refer to Supplementary Table 5. It is worthy to note that there is no intersection of significantly affected TFs between the two *BCL2* regulatory mutation blocks. For TFs with increased binding affinity changes, ETS1 and AHR::ARNT complex are predicted to be significant in BCL2 regulatory mutation block one and two, respectively. Nevertheless, for the negative modification of TF binding affinities (Fig. 5), many more patient-specific regulatory mutation blocks contain commonly altered TFs. For instance, both GR (NR3C1) and NHLH1 binding affinities are significantly decreased in BCL2 regulatory mutation block one across 7 FL patients. Binding affinities of ARNT, NFE2L2, FOXL1, FOXD3, MEIS2, MEIS3, and MYC are significantly reduced (Bonferroni corrected P < 0.05) in 75% (9 out of 12) of FL patients whom have SNVs in the BCL2 regulatory mutation block two. Though ARNT binding affinity is predicted to be significantly affected in BCL2 regulatory mutation blocks, it does not bind to ±1000 bp of BCL2 TSS according to the ReMap database. In summary, the negative change of TF binding affinities happened more frequently than that of the positive ones in FL.

### Verification of BayesPI-BAR2 on a validation cohort of 22 FL patients

The results presented so far are based on the computational prediction from a test cohort of 14 FL patients. Though the BayesPI-BAR2 pipeline is designed to remove potential biases from *in silico* predictions by performing a stringent statistical test against random background mutations, the robustness of the prediction does depend on the input data such as the quality of called somatic mutations, the precision and diversity of input PWMs, and patient sample size. Therefore, we repeated the same analysis on a validation cohort (22 FL patients) to assess the reproducibility of aforementioned results. The mutation data of 22 new FL patients were downloaded from ICGC public data release, which contain whole-genome somatic mutations (~181135) called by SAMtools^40^. In the same gene regulatory regions (±1000 bp to TSS), there are more mutations in this new cohort (22 FL patients; ~2953) than that of the test cohort (14 FL patients; ~795). That is because the called mutations for the test cohort are based on an intersection of two programs. By applying the first step of BayesPI-BAR2 pipeline (e.g., MuSSD) on the validation FL cohort, we detected almost the same mutation blocks in the promoter regions of *BCL6* and *BCL2* as that of the test cohort. For example, in Supplementary Figure 8, sixty-nine of seventy-four mutations from the test cohort are located inside the *BCL6* and *BCL2* mutation blocks of the validation cohort.

Since the identified regulatory mutation blocks at *BCL6* and *BCL2* are largely overlapping (Supplementary Figure 8) between the validation and test cohort, we repeated the second step of BayesPI-BAR2 pipeline at validation cohort mutations located in blocks defined by the test cohort (Fig. 2), to investigate potential correlations between the TF binding affinity changes and regulatory mutation blocks. The results are shown in Supplementary Table 6, where the predicted significantly affected TFs from the test and the validation cohorts at three mutation blocks are displayed. TBP in the *BCL6* block, and GR, ARNT, NFE2L2, and FOXL1 in the two *BCL2* blocks are significantly affected in both cohorts. Nevertheless, there are predicted TFs that only appear in one of the cohorts. This may be caused by differences in numbers of called mutations and sample sizes between the test and validation cohorts. For example, a patient from the test cohort (14 FL) has 2.6 mutations per block on average, while a patient from the validation cohort (22 FL) has 7.4. This can result in more TFs predicted to be affected by mutations of validation cohort than that of test cohort.

### Chromosome Translocation in *BCL6* and *BCL2*

**Figure 6:**
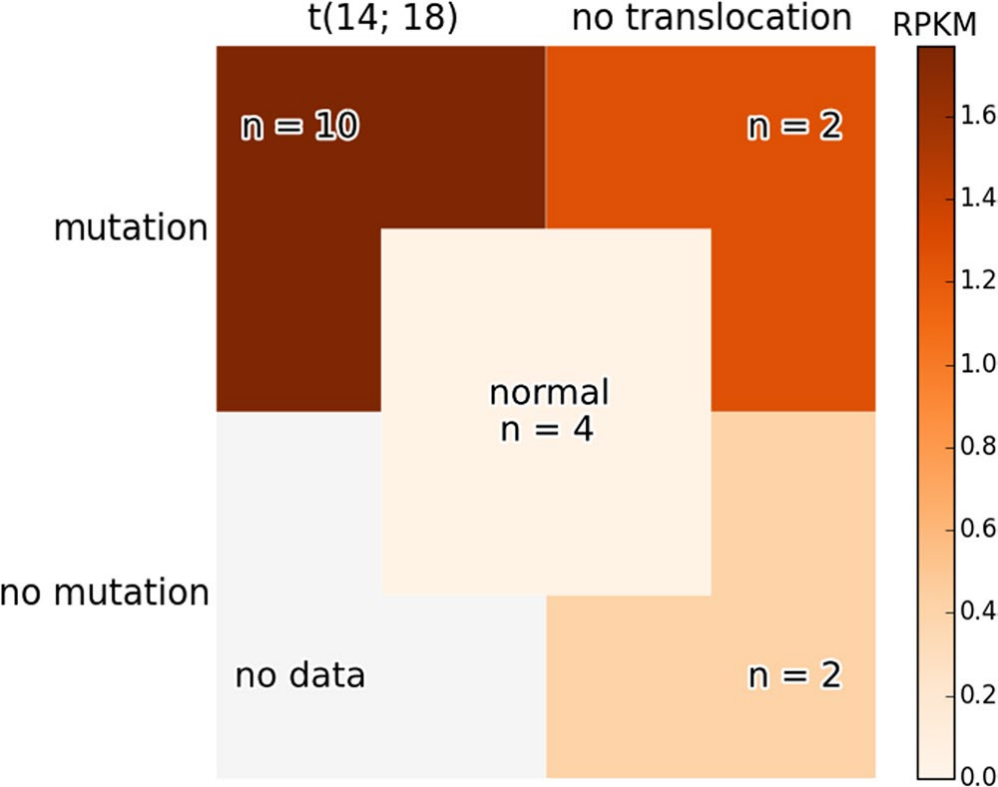
*BCL2* expression dependency on mutations. The heat-map shows median *BCL2* expression level (RPKM) for groups carrying combinations of *BCL2* regulatory block SNVs and t(14; 18) translocation. The median expression level of the four normal GCB samples is shown in the middle for comparison.

*BCL6* is reported to have frequent translocations in FL^41^. We tested all samples for *BCL6* translocations by DELLY2 and found only one case with *BCL6* moved into *IgH* locus (Supplementary Table 7, Supplementary Figure 9a). For *BCL2*, the t(14; 18) translocation is considered to be the main cause of elevated *BCL2* expression in FL^16^. We have presented evidence that SNVs in regulatory blocks near *BCL2* may affect expression as well. It was therefore of interest to study the relative contribution of these factors. To this end, we tested the 14 FL samples for presence of t(14; 18). This translocation was detected in 10 out of 14 samples (71%). As Supplementary Figure 9b shows, all *BCL2* breakpoints occur near the 3′ end, which means that the gene will carry regulatory mutation blocks with it if the translocation occurs. Supplementary Table 7 provides a summary of somatic mutations found for all patients. Figure 6 shows *BCL2* gene expression for three subgroups of patients, based on whether they have at least one SNV in a *BCL2* regulatory mutation block and whether they have a t(14; 18) translocation. t(14; 18) seems to increase *BCL2* expression, although not as strongly as the regulatory SNVs. The difference between *BCL2* expression of t(14; 18) positive samples (n = 10) and samples without t(14; 18) (n = 4) is not statistically significant, KS-test P < 0.19. However, the *BCL2* expression is significantly different between groups with a *BCL2* regulatory SNV (n = 12) and without (n = 2), KS-test P < 0.021 (Fig. 3b). Even in samples without the translocation, *BCL2* expression is elevated in FL compared to normal germinal center cells (KS-test P < 0.03). t(14; 18) and the regulatory SNVs appear to have an additive effect on the gene expression, although more data is needed to confirm this.

### Experimental Verification

**Figure 7:**
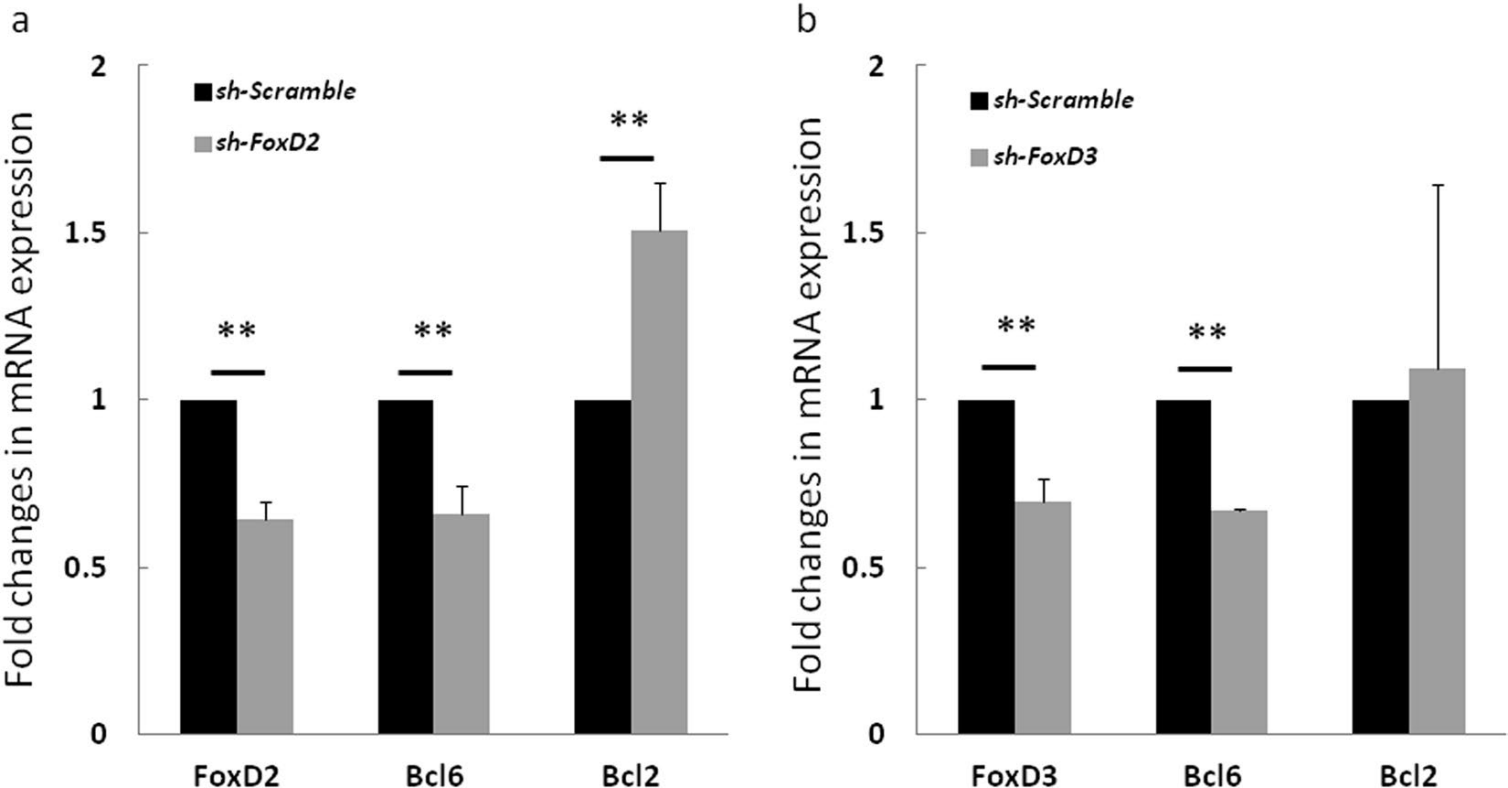
Down-regulation of *FOXD2* and *FOXD3* alters *BCL2* and *BCL6* gene expression. Effects of downregulation of *FOXD2* (**a**) and *FOXD3* (**b**) on *BCL2* and *BCL6* expression in SUDHL4 cells. (**a**) Knock down of *FOXD2* by shRNA decreased *BCL6* expression and increased *BCL2* expression in SUDHL4 cells in comparison to control (scramble shRNA). (**b**) Knock down of *FOXD3* decreased *BCL6* expression in comparison to scramble shRNA. **P < 0.01 (two-tailed t-test).

In order to verify our predicted novel TF-gene interactions that are disrupted by non-coding SNVs in FL, we randomly selected four genes (*ARNT*, *GR*, *FOXD2*, *and FOXD3*) from Figs 4 and 5 to perform the knockdown experiment. Expression of two of the genes (*FOXD2* and *FOXD3*) was transiently down regulated by shRNA in human B lymphocyte SUDHL4 cells. Therefore, these two genes were finally selected to verify the effect of the regulatory SNVs on gene expression of *BCL2/BCL6*. The shRNA knockdown resulted in 36% and 31% reduction of mRNA expression of *FOXD2* and *FOXD3*, respectively, in comparison to scramble control shRNA. Correspondingly, down-regulation of *FOXD2* led to a 35% reduction in transcription of *BCL6* (two-tailed t-test, P < 0.01) and an increased *BCL2* expression (1.5-fold; P < 0.01), Fig. 7a. Similarly, down-regulation of *FOXD3* resulted in 33% reduction of *BCL6* expression (P < 0.01) but a minor increase of *BCL2* expression, Fig. 7b. Thus, these experiments showed that knockdown of *FOXD2* or *FOXD3* modulates *BCL2* or *BCL6* in human B lymphocyte cells according to our computationally predicted effects of the SNVs on TF binding.

## Discussion

A genome-wide sequencing experiment may obtain ~10000 to 20000 SNVs^42^, which presents a very challenging task for any *in silico* method that attempts to predict functional non-coding mutations. To identify TF-DNA interactions that are affected by these non-coding SNVs is especially challenging because there are thousands of TFs in the human genome. In this study, we focused on promoter regions (±1000 bp to TSS) of known protein coding genes, ignoring many SNVs that may be located in other functional regulatory regions (e.g. enhancers and insulators) since it is relatively easy to find a target gene for a functional SNV at a promoter region, compared to an SNV positioned in a distant regulatory region. In this way, we obtained 795 reliably called SNVs from genome-wide sequencing of a test FL cohort (14 patients). To predict functional non-coding mutations among the SNVs, we developed a new integrated approach BayesPI-BAR2 (Fig. 1). In the first step of BayesPI-BAR2 pipeline, we used MuSSD algorithm to identify putative functional mutations from the patients. The MuSSD algorithm integrates the spatial and sample distribution of SNVs across multiple FL patients with the tumor-normal paired differential expression of putative target genes, to predict functional non-coding mutations. This analysis reduced the number of potentially interesting non-coding mutations to 76. A maximum distance between any two SNVs in a valid regulatory mutation block was chosen to be 30 bp in the final algorithm. The same result was obtained with a maximum distance of 50 bp. Of interest, the 76 SNVs are distributed across promoter regions of three genes (*BCL6*, *BCL2*, and *HIST1H2BM*; Fig. 2 and Supplementary Figure 1), where only 2 SNVs are near *HIST1H2BM* but 34 SNVs locate at a main regulatory mutation block (~500 bp; Fig. 2a) of *BCL6* and the other 40 SNVs spread to two main regulatory mutation blocks (~350 bp; Fig. 2b) of *BCL2*. The two main regulatory mutation blocks of *BCL2* are positioned in two known promoter regions of *BCL2*, which function differently between the normal germinal center cells and follicular lymphoma cells^43^. Notably, all 14 FL patients contain at least one SNV in one of the predicted main regulatory mutation blocks in either *BCL2* or *BCL6*. Particularly, in a validation FL cohort (22 patients), we found almost the same three mutation blocks (Supplementary Figure 8) in the promoter regions of *BCL6* and *BCL2*, respectively.

Since more than 97% (~74 SNVs) of predicted putative functional regulatory SNVs of FL are located at *BCL2* and *BCL6* promoters (±1000 bp to TSS), we attempted to predict TF-DNA interactions that may be affected by these SNVs. The new BayesPI-BAR program was first used to analyze TF binding to 61 bp long DNA sequences centered on individual SNVs. We found that the predicted SNVs are more frequently linked to the negative TF binding affinity changes than that to the positive ones; the binding affinities of TBP and CEBPB are often decreased for a given SNV at *BCL6* promoter; binding affinities of the STAT protein family are most frequently altered due to a SNV at *BCL2* promoter. However, we did not find any TFs that are affected by more than 30% of SNVs at either *BCL2* or *BCL6* (Supplementary Figures 4 and 5). Next, using the newly developed BayesPI-BAR2 pipeline, we analyzed the frequency of altered TF binding in the main regulatory mutation block, using patient-specific alternative DNA sequences (~500 bp and ~350 bp) at promoters of *BCL6* and *BCL2*, respectively (Fig. 2). Subsequently, statistical significance tests of TF binding affinity changes were performed, comparing the patient-specific mutation blocks to randomly generated ones.

Among the most significantly affected TFs, we found that binding affinity of TBP and members of the FOX protein family are decreased, by sequence variations of the *BCL6* regulatory mutation block, in 9 out of 10 FL patients with mutations in this block (Fig. 4). For *BCL2*, the two main regulatory mutation blocks are located in two separate promoters (Fig. 2b), and the TFs affected by these mutations are also split into two groups (Fig. 5): GR and ETS1 are often affected at *BCL2* promoter one, while the FOX protein family, NFE2L2, MEIS2, MEIS3, MYC, and the AHR::ARNT complex are strongly affected at *BCL2* promoter two. The BayesPI-BAR2 and other similar programs based on PWMs as the affinity models do have an inherent weakness: a TF may have multiple alternative PWMs, which makes its DNA binding affinity uncertain. This uncertainty propagates to the end result of the computation, which may cause the same TF to appear in both positively and negatively affected TF ranking list. For example, in the second mutation block of *BCL2* (Fig. 2b, P2; Fig. 5), ARNT and the AHR::ARNT complex have similar but distinct PWMs (Supplementary Figure 10), are predicted to be both positively and negatively affected ones. Nevertheless, according to the *in vivo* experiments that reported in ReMap, ARNT usually does not bind to ±1000 bp of BCL2 TSS, and the predicted negative binding affinity change may be neglected.

Based on an *in silico* study of genome-wide sequencing data of both test (14 patients) and validation (22 patients) FL cohorts, we have identified one and two main regulatory mutation blocks near the TSS of *BCL6* and *BCL2*, respectively (Fig. 2 and Supplementary Figure 8). In the regulatory mutation block of *BCL6* (Fig. 2a), only 12 of 34 SNVs are reported in dbSNP^44^. For *BCL2*, none of 40 SNVs in the two main regulatory mutation blocks (Fig. 2b) are listed in dbSNP database. Twenty-six out of forty *BCL2* regulatory SNVs are located in the promoter regions of two genes (*BCL2* and *KDSR*) based on ANNOVAR analysis^45^ (i.e. their distance to the TSS of *BCL2* is ~1000 bp, and the distance to the TSS of *KDSR* (*FVT-1*) is ~6000 bp, see Supplementary Figure 9). Since these 26 SNVs are located more than 1Kbp away from the TSS of *KDSR*, *KDSR* is not considered as a putative target gene by the MuSSD algorithm. However, similar to *BCL6* and *BCL2*, *KDSR* is differentially expressed between tumor and normal paired FL samples (KS-test P < 0.001). Thus, our predicted *BCL2* regulatory mutation blocks (Fig. 2b) may affect the regulation of other genes, apart from *BCL2* (e.g., *KDSR*), which are known to have a role in FL^46, 47^. Additionally, for *BCL2*, not only two main regulatory mutation blocks were found in the two *BCL2* promoters (Fig. 2b), but an overlap between the *BCL2* promoters and two known super-enhancers in lymphoma cell lines was also detected^36^. Of interest, the two main regulatory mutation blocks of *BCL2* are also positioned in the regions that are differentially methylated (DMRs) between lymphoma and germinal center B cell controls according to an earlier publication^24^. Methylation may add to differential binding of TFs. It is also worthy of note that there are frequent somatic hypermutations (SHM) of the 5′ non-coding region of the BCL6 in both B-cell lymphoma^35^ and normal germinal center B cells^48^. The three discovered putative functional regulatory mutation blocks may be related to SHM. Mistargeted SHM in a gene regulatory region can dysregulate gene expression and contribute to lymphomagenesis^49^, such as at *BCL6* and *BCL2* ^50^ in FL.

The relative effect of non-coding SNVs on *BCL2* expression is stronger than that of t(14; 18) as predicted by our analysis (Fig. 6). This may be indicated by the fact that 12 FL patients have SNVs in one of the regulatory mutation blocks in *BCL2* (Fig. 2), but only 10 cases show the translocation (Supplementary Table 7). Another more convincing reason is that the BayesPI-BAR2 predicted TFs, with altered binding affinities in patient-specific regulatory mutation blocks of *BCL2*, can explain the differential gene expression between FL and GCB control samples. For example, gain of ETS family binding sites has been previously reported as a cause of increased transcriptional activity of the *TERT* gene in melanoma^51^. *BCL2* gene is known to be regulated by ETS1^52^, and an over expression of ETS1 in human endometrial adenocarcinoma HEC-1-A cells can result in an increase in BCL2 protein expression^53^. Therefore, a similar effect may exist in follicular lymphoma, where ETS1 activates BCL2 expression after increasing its binding affinity at the *BCL2* regulatory mutation block one (Fig. 2b, P1; Fig. 5). GR (Glucocorticoid Receptor, NR3C1) has diverse roles in the immune system^54^, including promotion of the immunosuppressive effect of glucocorticoids (GC). Upon GC stimulation, GR indirectly down regulates *BCL2* expression^55^. Thus the reduction of this regulatory activity by the mutations may contribute to the increased *BCL2* expression as well.

Since AHR::ARNT complex increases *BCL2* expression under some conditions^56^, the positive change of its binding affinity at BCL2 promoter two (Fig. 2b, P2; Fig. 5) may cause gene dysregulation in FL. The role of other affected TFs is difficult to quantify due to lack of evidence, though most of them are reported to be involved in follicular lymphoma (e.g., NFE2L2^57^, MYC^58^, the FOX family^59^). In addition to the aforementioned direct binding effects, SNVs in the regulatory sequences of *BCL6* causing *BCL6* down regulation may also increase *BCL2* expression. The binding of TBP and the forkhead family of transcription factors is negatively affected by SNVs in the main regulatory mutation block of *BCL6* (Figs 2a and 4). TBP is related to transcription factor activity at RNA polymerase II core promoter proximal region, and the loss of TBP binding at *BCL6* promoter region may cause *BCL6* down regulation. Some of forkhead family of TFs (e.g., FOXO) are known to positively regulate *BCL6* gene through the FOXO signaling pathway^60^. Thus, a decrease of forkhead protein binding to the *BCL6* promoter may also result in lower expression of *BCL6*. Since BCL6 represses *BCL2* in normal GCB cells, *BCL6* down regulation will have a positive effect on *BCL2* expression^46^. This hypothesis is consistent with our *FOXD2* or *FOXD3* knockdown experiment in human B lymphocyte SUDHL4 cells (Fig. 7).

The current study investigated the hypothesis that changes of gene expression of *BCL2* and *BCL6* are a consequence of mutations within the regulatory mutation blocks in the promoters. The proposed new integrative genome sequence analysis method, BayesPI-BAR2, is designed to identify functional non-coding mutations in the genome. It is not able to consider the potential impact of mutations in the coding region. Mutations within other genomic regions, such as coding and untranslated regions (UTRs), or translocations, can also contribute to dysregulation of gene activity in disease. For FL, frequent coding sequencing mutations was found in *BCL2* ^50^, as well as mutations in genes involved in epigenetic regulation and chromatin modification such as *MLL2*, *CREBBP*, and *EP300* ^61^. In the future, a combination of the proposed genome-wide sequencing analysis pipeline and numerous coding sequencing mutation analysis tools will significantly improve the understanding of DNA sequencing variation in cancer.

In conclusion, by applying an integrated analysis of genome-wide sequencing data of FL, we discovered three previously unknown regulatory mutation blocks at the promoter regions of only two genes, *BCL6* and *BCL2*, known to be important oncogenes in FL. The finding of these mutation blocks in genes that are well-known to be important in FL, is an indication that the discovered mutation blocks are involved in the abnormal regulation of these genes. The results suggest that t(14; 18) translocation and the regulatory SNVs in the promoters of *BCL6* and *BCL2* appear to have an additive effect on the gene expression. The proposed new integrative analysis is not only useful for identifying functional non-coding mutations based on whole genome sequencing data, but also can predict novel TFs whose binding is disrupted by non-coding mutations in cancer. A future plan is to extend this study to explore unknown driver mutations in long distance region (e.g., enhancer-promoter interaction) by considering 3D chromosome structure^62, 63^.

## Materials and Methods

### Genome-wide sequencing data and RNA-Seq data analysis

We obtained genome-wide sequencing data of 14 tumor-normal paired FL patients, reported in an earlier publication^26^, by getting access to controlled data from ICGC. All aligned BAM files of tumor-normal paired whole-genome sequencing data and the corresponding RNA-Seq data of tumor samples were downloaded from European Genome-phenome Archive^64^ (http://www.ebi.ac.uk/ega/) under accession numbers EGAD00001000645 and EGAD00001000355. RNA-Seq data of four control samples (Germinal center B-cell - GCB) from healthy people were downloaded from GEO database under accession number GSE45982^65^. All sequencing data were aligned to hs37D5, a variant of GRCh37 human genome assembly used by the 1000 Genomes project^27^. Here, mutations were called by using Strelka^28^ and MuTect^29^ with default parameters, respectively. For Strelka, genome-wide mutations were called. For MuTect, only mutations located within ±10000 bp to the transcription start sites (TSS) were called because of long waiting time for genome-wide local realignment in the MuTect. An intersection of mutation calls from both programs was used in further data analysis for each patient^14^. From mutations called by these programs, we only consider SNVs. For identifying the transcripts of all protein-coding genes, we used gene annotation from the GENCODE^32^ (v19), and the promoter regions defined as ±1000 bp to the TSS of protein-coding genes. Gene expression levels, reads per kilobase of exon model per million mapped reads (RPKM) of RNA-Seq experiments, were computed by applying the featureCounts^66^ and in-house Python code on aligned BAM files.

### BayesPI-BAR2 pipeline

In the first step of BayesPI-Binding Affinity Ranking 2 (BayesPI-BAR2) pipeline (Fig. 1), we group mutations from one patient into blocks for predicting their joint effects on TF binding: for example, several mutations may occur inside the same TF binding site, disrupting it stronger than that if they occurred individually. Only regions that have mutations from multiple patients are considered as mutation blocks, because regulatory effects that are recurring among the patients may be important for disease. Subsequently, mutation blocks close to the TSS of known genes, where the genes are differentially expressed between patients and normal control samples, will be further investigated by BayesPI-BAR2 analysis.

At the second step of BayesPI-BAR2 pipeline, we use an updated version of published BayesPI-BAR program^12^ to measure how a variant affects affinity of a TF. In the new program, several enhancements to biophysical modeling of protein-DNA interactions^33, 67^ were made: the shifted differential binding affinity (δdbA) scores from BayesPI-BAR are normalized to a common scale (e.g., zero mean and unit standard deviation) across all mutations before generating TF ranking for each mutation separately; the significance of each δdbA is estimated from the overall distribution of δdbA in the calculation; the amount of computation is reduced by ~70% thanks to an early stopping functionality in the BayesPI2+ random background sampling (the calculation stops when the TF binding affinity to DNA sequence is similar to that of background sequences); BayesPI-BAR can now distribute jobs across several computer nodes, increasing the level of parallelization. The new BayesPI-BAR has ~5% improvement in the prediction accuracy and reduced overall wall-clock time from several hours to ~15 minutes, when it is tested on the previously published 67 known regulatory mutations^12^.

In the BayesPI-BAR2 pipeline, we use δdbA values computed by BayesPI-BAR. The computed δdbA of each TF can be: positive, indicating increased TF-DNA binding due to the mutation; negative, indicating a disrupted binding site; or zero, indicating no discernible affinity change. Each patient’s mutations affect TF-DNA binding (δdbA) in a particular way. Some of them may be random, while others may cause a gene regulation disturbance that undergoes positive selection because it is beneficial for the tumorigenesis. We assume that the latter will show as a shift in the distribution of δdbA in patient samples. To evaluate the significance of such a shift, we compare a set of δdbA from patients to a set of δdbA obtained from randomly generated background mutations for the same TF, by using the Wilcoxon rank-sum test. In this way, TFs whose binding affinity changes are strongly associated with detected functional regulatory mutation blocks can be identified.

To test whether a TF is significantly affected by SNVs in the patient dataset, we compare patient-specific δdbA values (in a given regulatory mutation block) to a background distribution of δdbA values (in a set of randomly generated mutation blocks). First, the background mutation blocks were extracted randomly from 1000 bp upstream of TSS of all known genes (20376 regions in total), with the same sequence length and the number of mutations as the block being tested. The gene carrying the patient-specific mutation block is not included in the random background selection. The reference sequence of each background mutation block is taken from hs37D5, and the corresponding alternative sequence is generated by randomly altering nucleotides in the selected region. Thus, for each given TF and a regulatory mutation block of 14 FL patients, we can obtain 20375 δdbA values to represent a background δdbA distribution by applying BayesPI-BAR on randomly generated mutation blocks. Then, Wilcoxon rank-sum test is used to compare the distribution of δdbA values between the patients and the randomly generated mutation blocks. Bonferroni-corrected P values are used for final significance selection. The proposed statistical test considers both the strength of TF binding affinity change and the recurrence of δdbA values across the samples. For each TF, the significance test is repeated three times.

### *In silico* calculation of TF binding affinities and ranking of TFs affected by called mutations

In order to predict putative TFs that may be affected by called mutations of selected gene promoter regions, we downloaded 2065 PWMs representing about 617 unique human TFs from an earlier paper^68^, where 1772 of PWMs that come from reliable sources (labeled by “known” set) were considered in the final prediction. Then, our previously developed biophysical modeling of protein-DNA interactions^13^ – BayesPI2^33^ – was applied to estimate the *in silico* TF binding affinity at DNA sequences, and a newly upgraded BayesPI-BAR^12^ program (Supplementary Methods) was used to rank putative TFs that may be affected by the called mutations. We made two types of DNA sequences for the test. One is 61 bp long DNA sequences centered at each SNV (which we call individual SNV sequence because only one mutation is included in the sequence), and the other is the patient-specific alternative DNA sequence at a regulatory mutation block. A regulatory mutation block is a genome region 350–500 bp long containing many recurrent SNVs, defined by the MuSSD algorithm (see below). For each patient, all his/her SNVs located in a regulatory mutation block will be included in the alternative DNA sequence, which spans the entire block. This sequence is called patient-specific alternative DNA sequence or patient-specific regulatory mutation block. The corresponding reference DNA sequences for each patient will be taken from the hs37D5 human genome assembly.

### Identification of putative target genes of called regulatory mutations

We designed a novel algorithm (Mutation filtering based on the Space and Sample Distribution - MuSSD; Supplementary Methods) to remove non-informative mutations at the gene promoter regions, for 14 tumor-normal paired FL patients. First, we assumed that the functional non-coding mutations are physically adjacent to each other at a predefined genomic region. Correspondingly, if a distance between the two distinct mutations, belonging to either two patients or to the same patient, is smaller than 30 bp, then the two mutations will be merged together to become a potential regulatory mutation block. Such computation is performed recurrently, until all called mutations in the gene promoter regions from the 14 FL patients are assigned to mutation blocks according to their spatial distributions. Subsequently, we hypothesized that a functional regulatory mutation block had to show at least two mutations derived from two different patients. Thus, any mutation blocks that do not meet this requirement will be removed by MuSSD. Finally, for all promoter regions that contained at least one functional regulatory mutation block as defined above, we investigated the differential expression of corresponding genes (RPKM) between the tumor (FL) and normal control (GCB) samples, by using a two-sample Kolmogorov-Smirnov goodness-of-fit hypothesis test (KS-test)^33^. Using this test, a P-value smaller than 0.05 identifies the putative target gene for a regulatory mutation block.

### Differential gene expression analysis with baySeq

It is well known that simple RPKM based analysis of differential gene expression may be biased^69^. Thus the differential gene expression analysis was repeated using baySeq^34^, a tool designed for this task. RNA-Seq raw counts of known GENCODE protein-coding genes, except for mitochondrial ones, were used as input (19176 genes in total). A comparison between 4 GCB and 14 FL samples was carried out by baySeq, which uses Bayesian analysis to infer probabilities of differential expression of each gene and computes the false discovery rate (FDR). Here, a gene with FDR < 0.05 is considered as differentially expressed between tumor and normal samples.

### Filtering TFs from BayesPI-BAR2 predicted list by gene expression

Frequently, the expression level of a gene is used to judge whether the corresponding protein or RNA product is functional or not^70^. We assumed that if a TF is not expressed in a cell, then changes to its binding affinity due to DNA sequence variations do not affect gene regulation. Thus, a TF was removed from BayesPI-BAR2 predicted list if there was no expression of the corresponding gene, or the expression was extremely low (at the level of experimental background noise). To remove these TFs from the final prediction, we computed median RPKM for the corresponding genes in 14 FL patients’ RNA-Seq data, according to normalized counts from baySeq. Previously it was determined^70^ that RPKM of ~0.03 is the optimal threshold for distinguishing lowly expressed genes from experimental background noise. In this way, ~30% of TFs with RPKM < 0.03 are removed from TF ranking list that generated by the new BayesPI-BAR2 pipeline.

### Chromosome Translocation Analysis

We tested all FL samples for the presence of the chromosomal translocation t(14; 18). The program DELLY2^71^ was used to call translocations in tumor and normal samples, which were then filtered to retrieve somatic mutations only. We checked the presence of translocations near *BCL2* gene (TSS ± 5Mbp) with a corresponding location on chromosome 14, and also near *BCL6* gene (TSS ± 500Kbp) with any corresponding location. A few of translocations found this way are marked as low quality by DELLY2, which means that the number of reads is low, or the mapping quality is low. However, this calculation is based on uninformative prior probability of mutations. In FL, the prior probability of t(14; 18) is high, therefore the low quality translocations found in that region were considered to be true.

### Cell line and shRNA transfection

Human B lymphocyte line SUDHL4 was obtained from the American Type Culture Collection (ATCC, CRL-2957TM). The cells were maintained in RPMI-1640, supplemented with 20% fetal bovine serum, 2 mM Glutamine, penicillin/streptomycin in a humidified incubator with 5% CO_2_ at 37 °C. Fresh medium was added every two days and the cells were split at the ratio of 1:5. The FOXD2 and FOXD3 shRNA lentiviral particles and control particles were purchased from Santa Cruz Biotechnology. For viral infection, the SUDHL4 cells were placed in 6-well plate at 1 × 106 cells/well supplemented with 8 ug/ml polybrene (Santa Cruz Biotechnology) and 20 ul of either control, FOXD2, or FOXD3 shRNA particles. The cells were centrifuged at 2,000 rpm for 2 hrs at 37 °C. After centrifugation, the cells were returned to humidified incubator for continuing culture. Forty-eight hours later, the cells were split and 0.4 ug/ml puromycin dihydrochloride (Santa Cruz Biotechnology) was added into the cells for selection. The shRNA expression efficiency was determined by quantitative real-time PCR (qPCR).

### RNA extraction and quantitative RT-PCR

Total RNA from SUDHL4 cells was isolated with the RNeasy Mini Kit (Qiagen) according to the manufacturer’s instructions. RNA concentration was determined by a spectrophotometer (Nano Drop® 1000) and reversely transcribed using the high Capacity Reverse Transcription Kit (Applied Biosystems). qPCR was performed with a StepOnePlus Real-Time PCR System (Applied Biosystems) using the Power SYBR green PCR Master mix (Applied Biosystems). Oligonucleotides used were as follows: β-Actin, forward 5′- GTTACAGGAAGTCCCTTGCCATCC, reverse 5′- CACCTCCCCTGTGTGGACTTGGG; FoxD2, forward 5′-GGGAGAGGGGAGGGAGAAAT, reverse 5′-GAGTCTCTGTGGAAACGGCA; FoxD3, forward 5′-CGCCACAACCTCTCACTCAA, reverse 5′-GTCCAGGGTCCAGTAGTTGC; Bcl2, forward 5′-CTGCACCTGACGCCCTTCACC, reverse 5′-CACATGACCCCACCGAACTCAAAGA; Bcl6, forward 5′-CTGCAGATGGAGCATGTTGT, reverse 5′-TCTTCACGAGGAGGCTTGAT. Samples were normalized to housekeeping gene β-Actin and fold change of expression levels was determined from the difference in ΔCT values.

### Data availability

The datasets generated during and/or analysed during the current study are not public available due to ICGC controlled data access policy but are available from the corresponding author on reasonable request.

## Electronic supplementary material

supplementary information
