# Supplementary material for: Integrative whole-genome sequence analysis reveals roles of regulatory mutations in BCL6 and BCL2 in follicular lymphoma
Source: Sci Rep. 2017 Aug 1;7:7040. doi: 10.1038/s41598-017-07226-4 (PMC5539289; doi:10.1038/s41598-017-07226-4)
Supplement: Supplementary file 1 — supplementary information [file 41598_2017_7226_MOESM1_ESM.pdf]

Integrative whole-genome sequence analysis reveals roles of regulatory mutations in *BCL6* and *BCL2* in follicular lymphoma

Kirill Batmanov, Wei Wang, Magnar Bjørås, Jan Delabie, and Junbai Wang

Supplementary information

Supplementary Materials and Methods

MuSSD

Mutation filtering based on the Space and Sample Distribution (MuSSD) algorithm is designed to find highly mutated DNA regions that may be important for gene regulation, and is based on the following assumptions:

- Two distinct mutations with a distance smaller than a predefined value (here 30bp), likely disrupt the same regulatory element, and can be joined together into one regulatory mutation block.
- A regulatory mutation block will be considered as important for a phenotype (e.g., a disease), if it contains mutations from more than one sample (or patient) and is located close to a gene promoter (a 1kb region centered on the transcription start site of a gene).
- A regulatory mutation block will be further considered as functional towards the nearest gene, if it disrupts the nearest gene activity. Such disruption is defined as a significant differential gene expression between the tumor and normal control samples.

BayesPI

BayesPI[1] is a biophysical model of transcription factor (TF)-DNA interaction, which takes into account the chemical potential (or concentration) of a TF in a cell nucleus. The TF-DNA binding probability is

$$P(S,w,\mu)=\sum_{i=0}^{N-M}\frac{1}{1+\exp(\sum_{j=1}^{M}\sum_{k=1}^{4}w_{j,k}S_{i+j,k}-\mu)}$$

where $S_{i,k}$ = 1 if the DNA sequence has nucleotide k (one of A, C, G, T) at position i, otherwise $S_{i,k}$ = 0; $w_{j,k}$ is the binding energy for nucleotide k at position j of the TF binding motif; N is the sequence size; M is the motif size; μ is the chemical potential. To distinguish the direct binding from indirect TF-DNA interaction, a differential binding affinity is used:

$$dbA(S,w,\mu,R) = P(S,w,\mu) - \frac{1}{R}\sum_{i=1}^{R} P(\text{shuffle}(S),w,\mu)$$

where R and `shuffle`(S) are the number of random permutations and a function used to randomly permutes the input sequence, respectively. Usually, R = 10000 is sufficient to estimate the differential binding affinity dbA. Additionally, a P-value to estimate the significance of a direct TF-DNA interaction is calculated:

$$P_{dbA}(S,w,\mu,R) = \frac{1}{R}\text{count}[P(S,w,\mu) > P(\text{shuffle}(S),w,\mu)]$$

where the `count[]` function calculates how many times the TF binding probability of the sequence S was higher than that of randomly shuffled sequences.

Major assumptions of BayesPI are:

- The binding probability (or affinity) of a TF to a DNA sequence is fully determined by the sequence information, the nucleotide preference of the TF, and the TF concentration.
- Nucleotide preference of a TF can be fully described by a 4 × M nucleotide TF binding energy matrix, where each nucleotide contributes to the TF binding probability independently.
- A direct TF-DNA interaction shall often have higher binding affinity at the input DNA sequence than that of the randomly shuffled sequences, which will be reflected in a positive *dbA* score and a small P_{dbA}.

BayesPI-BAR

BayesPI-Binding Affinity Ranking (BayesPI-BAR)[2] introduced a new score to assess the effect of DNA sequence variation to TF binding, which is called shifted differential binding affinity:

$$\delta dbA\left(S_{ref}, S_{alt}, w, \mu, R\right) = dbA\left(S_{ref}, w, \mu, R\right) - dbA(S_{alt}, w, \mu, R)$$

where S_{ref} and S_{alt} are the reference and alternate (mutated) DNA sequences, respectively. The binding energy matrices w can be obtained from common position specific weight matrices (PWM), which are available for many human TFs. However, the chemical potential μ is usually unknown. BayesPI-BAR computes *δdbA* scores for a predefined set of chemical potentials in a plausible range (here, μ = 0, -10, -13, -15, -18, and -20). Then these *δdbA* scores are integrated by using either a simple average method or a principal component analysis based approach. The final ranking of TFs affected by a mutation is based on the integrated *δdbA* score.

Major assumptions of BayesPI-BAR are:

- The set of available PWMs contains all important TFs that may be disturbed by the sequence variation in FL.
- TF-DNA binding affinity changes due to a given mutation can be estimated by scanning a DNA sequence with known TF PWMs where the TFs are assumed to directly interact with the DNA sequence.

Aberrant somatic hypermutation analysis

Following Rogozin and Diaz[3], we counted how many mutations in each block fall into the DGYW/WRCH somatic hypermutation (SHM) hotspot motif. In this motif: D stands for A, G, or T; Y is C or T; W is A or T; H is T, C or A; underlined is the mutation position; the second 4-mer is the reverse complement of the first. For each block, we first count the number *m* of motifs in it. $p = m$ / (block length) is the probability that a randomly chosen mutation will fall into a SHM hotspot motif under the null model of mutations being uniformly distributed across the block. Then we count the number *x* of patient mutations in this block that occur within SHM hotspot motifs. If *N* is the total number of patient mutations within this block, then $f = x / N$ is the observed frequency of patient mutations within this block which occur in SHM hotspot motifs. We calculate a two-sided binomial test P value with parameters *x*, *N*, and *p*. If it is significant at $P < 0.05$ and $f > p$, we conclude that the patient mutations occur within SHM hotspot motifs significantly more frequently than expected by the null model, and thus there is a contribution of aberrant SHM to the mutagenesis in this block.

Supplementary References

1. **Wang, J., and Morigen. (2009) BayesPI - a new model to study protein-DNA interactions: a case study of condition-specific protein binding parameters for Yeast transcription factors, *BMC bioinformatics 10*, 345.**
2. **Wang, J., and Batmanov, K. (2015) BayesPI-BAR: a new biophysical model for characterization of regulatory sequence variations, *Nucleic acids research 43*, e147.**
3. **Rogozin, I. B., and Diaz, M. (2004) Cutting edge: DGYW/WRCH is a better predictor of mutability at G:C bases in Ig hypermutation than the widely accepted RGYW/WRCY motif and probably reflects a two-step activation-induced cytidine deaminase-triggered process, *Journal of immunology 172*, 3382-3384.**

Supplementary Tables

Supplementary Table 1. Differential expression data by baySeq for 8 genes with mutation blocks near TSS.

Gene	Median normalized RNA-Seq counts for patients	Median normalized RNA-Seq counts for normal GCB	Likelihood	Ordering	FDR
BCL2	5201	132.5	0.99998	tumor>norm	2.92E-06
HIST1H2BM	227	11645.5	0.992846	norm>tumor	0.000832
BCL6	3665	12172.5	0.981954	norm>tumor	0.002094
IL4R	29122.5	43799	0.238319	norm>tumor	0.155891
TCL1A	96824.5	484782.5	0.032284	norm>tumor	0.362242
BIRC3	67831	75751	0.004251	norm>tumor	0.430282
IGLL5	83789.5	241856	0.000269	norm>tumor	0.448847
LTB	338798	362978.5	2.36E-05	tumor>norm	0.450083

The genes are ordered by likelihood of differential expression between tumor and normal groups. The last column is the false discovery rate for accepting the corresponding differential expression and all above it (analogous to P-value corrected for multiple hypothesis testing).

Supplementary Table 2. Nucleotide change statistics for mutation blocks near *BCL2, BCL6* and *HIST1H2BM*.

a) Single nucleotide change counts

Gene	C>A	C>G	C>T	T>A	T>C	T>G
BCL6	1	7	10	4	8	4
BCL2	2	6	15	3	9	5
HIST1H2BM	0	0	2	0	0	0

b) SHM motif occurrences

Mutation block	SHM hotspot motifs in block	Background probability of mutation in an SHM hotspot motif	Number of mutations	Number of mutations in an SHM hotspot motif	Frequency of SHM hotspot mutations	Binomial test P-value
BCL6	32	0.063	34	3	0.088	0.47
BCL2 block 1	28	0.08	14	6	0.43	0.00044
BCL2 block 2	21	0.065	26	5	0.19	0.025

These tables show statistics of mutations split by mutation block. **a**) Counts of either specified nucleotide changes or their complements in all SNVs within mutation blocks near TSS of the specified gene. **b**) aSHM statistical tests: we test whether the observed frequency of mutations occurring within SHM hotspot motifs DGYW/WRCH is different from expected under uniform-probability null model. Two-sided binomial test P values are given.

Supplementary Table 3. Number of TFs filtered by expression.

Gene	Affinity change direction	Sequence type	Total number of TFs in ranking	TFs with very low expression	% of TFs with very low expression
BCL6	Positive	individual SNVs	272	93	34%
BCL6	Negative	individual SNVs	220	70	31%
BCL6	Positive	patient-specific	85	32	37%
BCL6	Negative	patient-specific	62	20	32%
BCL2	Positive	individual SNVs	272	83	30%
BCL2	Negative	individual SNVs	222	63	28%
BCL2	Positive	patient-specific	141	39	27%
BCL2	Negative	patient-specific	115	29	25%

Counts of TFs in BayesPI-BAR rankings for each mutation block, and numbers of TFs filtered out because of very low expression.

Supplementary Table 4. Details of significant TFs affected by *BCL6* regulatory mutation block

Affinity change direction	PWM name	Gene name	Expression level	Is bound?	Affected patients	Bonferroni P value (minimum of 3 trials)	Bonferroni P value (maximum of 3 trials)
+	SMAD_1_ from_SMAD_ transfac_M00792	SMAD	Average	Bound	8 / 10	0.0085	0.0091
-	TATA_known3_ from_TBP_3_ from_TBP_ transfac_M00471	TBP	High	Bound	9 / 10	2.30E-08	3.50E-08
-	FOXD3_3_ from_FOXD3_ jolma_DBD_M410	FOXD3	Low	Not in ReMap	9 / 10	1.20E-07	1.30E-07
-	FOXD2_1_ from_FOXD2_ jolma_DBD_M408	FOXD2	High	Not in ReMap	9 / 10	1.80E-06	2.30E-06
-	IRX6_1_ from_Irx6_ bulyk_cell08-2623.2	IRX6	Average	Not in ReMap	8 / 10	2.50E-05	3.60E-05
-	MEIS2_2_ from_MEIS2_ jolma_DBD_M164	MEIS2	Low	Not in ReMap	8 / 10	0.00029	0.00038
-	HLX_1_ from_Hlx1_ bulyk_cell08-2350.1	HLX1	High	Not in ReMap	8 / 10	0.00043	0.00053
-	FOXC2_1_ from_FOXC2_ jolma_DBD_M405	FOXC2	Low	Not in ReMap	8 / 10	0.00046	0.00049
-	FOXC1_5_ from_FOXC1_ jolma_DBD_M404	FOXC1	Average	Not in ReMap	8 / 10	0.0088	0.01
-	TATA_known4_ from_TBP_4_ from_TBP_ transfac_M00980	TBP	High	Bound	8 / 10	0.017	0.02
-	FOXL1_2_ from_FOXL1_ jaspar_MA0033.1	FOXL1	Average	Not in ReMap	7 / 10	0.028	0.033
-	FOXC2_3_ from_FOXC2_ jolma_DBD_M407	FOXC2	Low	Not in ReMap	8 / 10	0.048	0.057

Data about each of PWM that is significantly affected by mutations near *BCL6*. The maximum and minimum rank-sum test P-values are given, out of three repetitions.

Supplementary Table 5. Details of significant TFs affected by *BCL2* regulatory mutation blocks

a) BCL2 promoter one regulatory block hg19:chr18:60986368-60986718

Affinity change direction	PWM name	Gene name	Expression level	Is bound?	Affected patients	Bonferroni P value (minimum of 3 trials)	Bonferroni P value (maximum of 3 trials)
+	ETS_known8_ from_ETS1_3_ from_ETS1_ jaspar_MA0098.1	ETS1	High	Bound	6 / 7	0.028	0.036
-	NR3C1_known17_ from_AR_9_ from_Ar_ jolma_DBD_M657	GR	Average	Bound	7 / 7	1.50E-34	2.50E-33
-	NR3C1_known4_ from_AR_2_ from_AR_ transfac_M00481	GR	Average	Bound	7 / 7	3.00E-19	2.10E-18
-	NR3C1_known13_ from_AR_6_ from_Ar_ jaspar_MA0007.1	GR	Average	Bound	6 / 7	1.00E-10	1.30E-10
-	NR3C1_known16_ from_AR_8_ from_AR_ jolma_full_M656	GR	Average	Bound	5 / 7	3.70E-08	9.00E-08
-	NR3C1_known15_ from_AR_7_ from_AR_ jolma_DBD_M655	GR	Average	Bound	5 / 7	8.80E-08	2.60E-07
-	NHLH1_4_ from_NHLH1_ jolma_DBD_M335	NHLH1	Average	Not in ReMap	6 / 7	0.0021	0.003

b) BCL2 promoter two regulatory block hg19:chr18:60988043-60988363

Affinity change direction	PWM name	Gene name	Expression level	Is bound?	Affected patients	Bonferroni P value (minimum of 3 trials)	Bonferroni P value (maximum of 3 trials)
+	AHR::ARNT_1_ from_AhR:Arnt_ transfac_M00235	AHR:: ARNT	Average	Not bound	8 / 11	5.90E-06	8.80E-06
-	ARNT_3_ from_Arnt_ jaspar_MA0004.1	ARNT	Average	Not bound	8 / 11	9.00E-09	4.50E-08
-	NFE2L2_3_ from_NFE2L2_ jaspar_MA0150.1	NFE2L2	Average	Not in ReMap	9 / 11	1.30E-07	2.40E-07
-	FOXL1_4_ from_FOXL1_	FOXL1	Average	Not in ReMap	9 / 11	1.10E-06	1.30E-06

	jolma_full_M423						
-	ARNT_1_ from_Arnt_ transfac_M00236	ARNT	Average	Not bound	8 / 11	4.30E-06	6.70E-06
-	MEIS3_3_ from_MEIS3_ jolma_DBD_M166	MEIS3	Average	Not in ReMap	9 / 11	6.70E-05	7.20E-05
-	MEIS2_4_ from_Meis2_ jolma_DBD_M168	MEIS2	Low	Not in ReMap	9 / 11	0.00017	0.0002
-	FOXD3_4_ from_FOXD3_ jolma_DBD_M411	FOXD3	Low	Not in ReMap	8 / 11	0.015	0.02
-	MYC_known4_ from_USF_2_ from_USF_ transfac_M00122	MYC	High	Bound	8 / 11	0.039	0.053

Data about each of PWM that is significantly affected by mutations near *BCL6*. The maximum and minimum rank-sum test P-values are given, out of three repetitions.

Supplementary Table 6. Comparison of significantly affected TFs in the three mutation blocks between the test cohort and the validation cohort of FL patients.

a) *BCL6* block

Direction	Test cohort (14 patients)	Validation cohort (22 patients)
Positive	SMAD	POU2F2
		SPIB
		SPI1
		HOXA10
		STAT4
Negative	TBP	MEF2
	FOXD3	CDX
	FOXD2	SOX18
	IRX6	SOX8
	MEIS2	SOX9
	HLX	RUNX1
	FOXC2	TBP
	FOXC1	EOMES
	FOXL1	SOX17
		SOX15

b) *BCL2* block P1 hg19:chr18:60986368-60986718

Direction	Test cohort (14 patients)	Validation cohort (22 patients)
Positive	ETS1	CUX1
		AP1
		IKZF1
		RFX5
		HOXA7
		EGR1
		PBX1
Negative	NR3C1	NR3C1
	NHLH1	IRF
		TEF
		FOXO3
		MAF
		POU2F2
		ESR2

c) *BCL2* block P2 hg19:chr18:60988043-60988363

Direction	Test cohort (14 patients)	Validation cohort (22 patients)
Positive	AHR::ARNT	GLIS2
		TFAP2
		ZNF740
Negative	ARNT	ARNT
	NFE2L2	FOXC1
	FOXL1	AHR::ARNT
	MEIS3	FOXL1
	MEIS2	FOXG1
	FOXD3	EGR1
	MYC	FOXD1
		NKX2
		FOXO4
		HEY2
		FOXO1
		NFE2L1::MAFG
		HLF
		ZEB1
		ZBTB14
		FOXJ2
		CLOCK
		GATA
		NFE2L2
		FOXP3
		FOXK1

a) Comparison for the *BCL6* mutation block. **b**) Comparison for the *BCL2* block **P1**. **c**) Comparison for the *BCL2* block **P2**. In each table cell the names of TFs significantly (rank-sum test, P-value < 0.05 after Bonferroni correction) affected by the mutations in the block are given. In a few cases a TF family name is given (e.g. GATA). TFs which are affected in both test and validation cohorts are marked with green.

Supplementary Table 7. Patient data summary.

**Sample ID**	***BCL6* SNV**	***BCL2* SNV**	**t(14; 18)**	**t(14; 18) in ICGC data**	***BCL6 trans-location***	**FL grade**	**Age at diagnosis**	**Survival time (days)**
4105105	Yes	Yes	Yes	Yes	No	I	40	516+
4121361	Yes	Yes	Yes (LQ)	No	No	I	74	123+
4134005	Yes	Yes	Yes	Yes	No	I	67	496+
4139696	Yes	No	No		Yes (LQ)	IIIb	70	306
4158726	Yes	Yes	Yes	Yes	No	I	48	510+
4159170	No	Yes	Yes	Yes	No	I	43	1615+
4160468	Yes	Yes	Yes (LQ)		No	II	62	2645
4170686	No	Yes	Yes		No	I	56	917+
4174905	Yes	Yes	Yes		No	I	72	1525+
4175837	No	Yes	Yes (LQ)		No	IIIa	74	420+
4177601	Yes	No	No		No	II	52	840+
4177987	No	Yes	No		No	II	71	203+
4188900	Yes	Yes	No	No	No	I	76	503+
4189200	Yes	Yes	Yes		No	I	51	1925+

Clinical data comes from the ICGC data portal. Structural variation data is available there for six FL samples, which is the same as our calculation except for ID 4121361 low quality (LQ) translocation detected by DELLY2. “+” in survival time means that the patient was alive at the time of the data collection.

Supplementary Figures

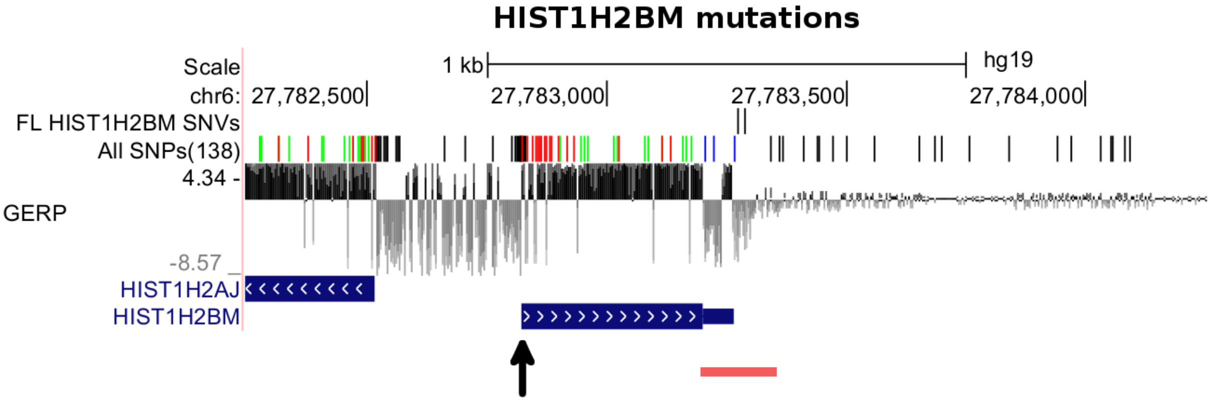

Supplementary Figure 1. Predicted regulatory mutation block in *HIST1H2BM* from a test cohort of 14 FL patients.

Genome Browser overview of the two HIST1H2BM mutations. For reference, all mutations from dbSNP and GERP conservation scores are given. Black arrow marks the TSS. Red bar represent the predicted regulatory mutation block

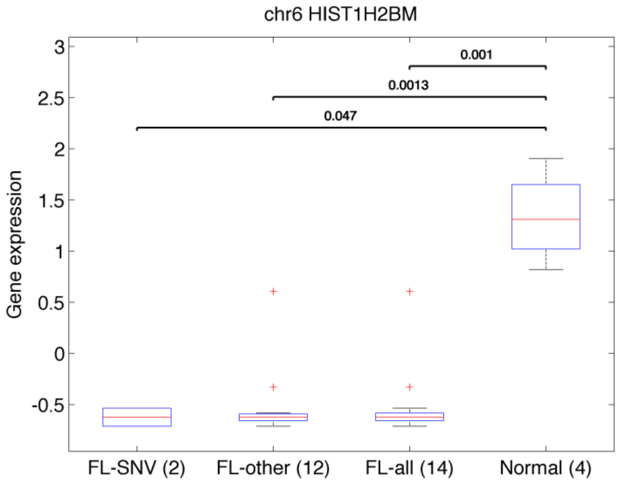

Supplementary Figure 2. Box plot of *HIST1H2BM* expression levels of 14 FL patients of test cohort and GCB control samples.

Here, FL-SNV represents FL patients with regulatory mutation blocks, FL-other represents FL patients without the regulatory mutation blocks, FL-all mean all 14 FL patients, Normal means GCB control samples. The number of FL patients/control samples in the category is given in parentheses. P-value of the significance of KS test for difference between gene expressions in two categories is given above the bar connecting corresponding categories.

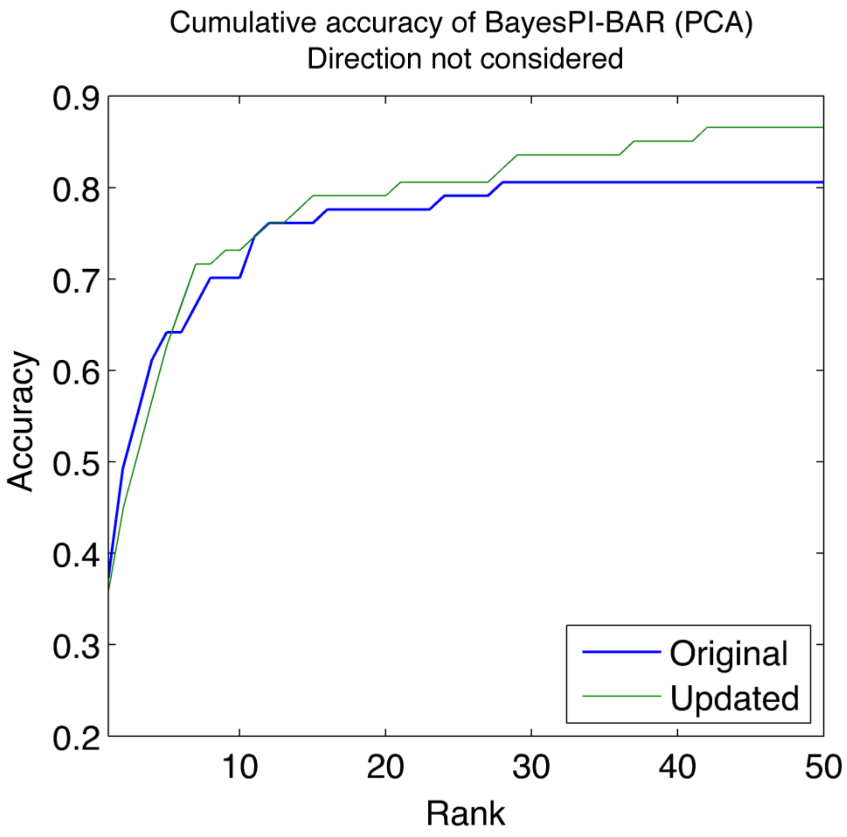

Supplementary Figure 3. Comparison of accuracy of original and updated new BayesPI-BAR on the 67 test mutations.

Cumulative accuracy plots for original and new BayesPI-BAR are shown. The X-axis represents the predicted rank, and the Y-axis shows the fraction of mutations for which the true TF appears with the corresponding or a lower rank. The positive or negative direction of TF binding affinity changes is not considered.

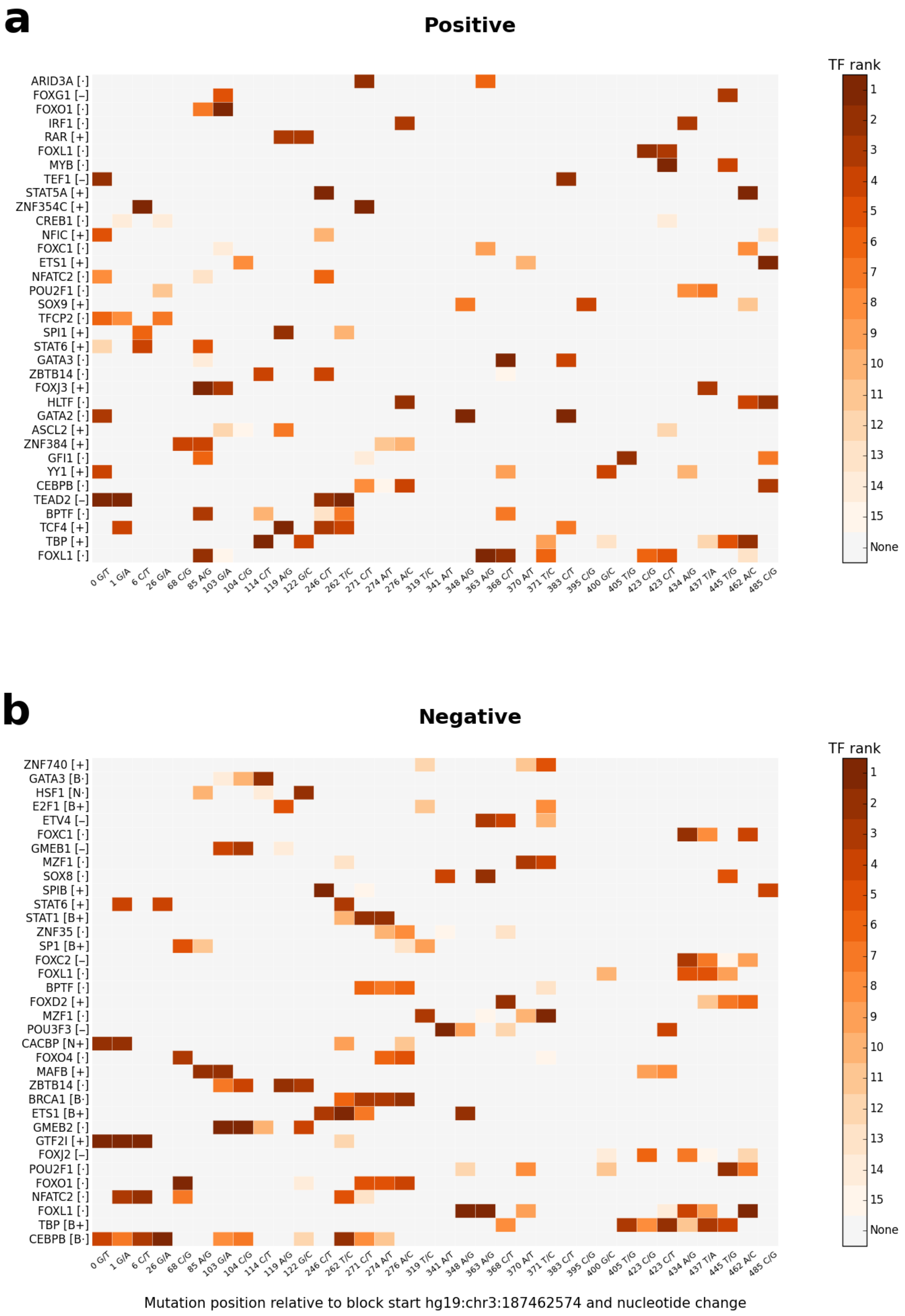

Supplementary Figure 4. TF affinity changes due to individual SNV at *BCL6* regulatory mutation block

a) Positive TF affinity changes due to individual SNV at *BCL6* regulatory mutation block; **b**) Negative TF affinity changes due to individual SNV at *BCL6* regulatory mutation block. Here, the row labels are the TF names, sorted by their frequency in FL patients. The color bar represents the rank order in the prediction: the lighter the color, the higher (less significant) the ranking. In square brackets inside row labels: “+” means that the TF is highly expressed (top 25%), “•” means average expression, “-“ means low expression (lower 25%), “B” means that the TF has a peak in TSS ± 1kb region in the ReMap dataset, “N” means that the TF is in the ReMap dataset but has no peak nearby. TFs with very low expression (RPKM < 0.03) were filtered out.

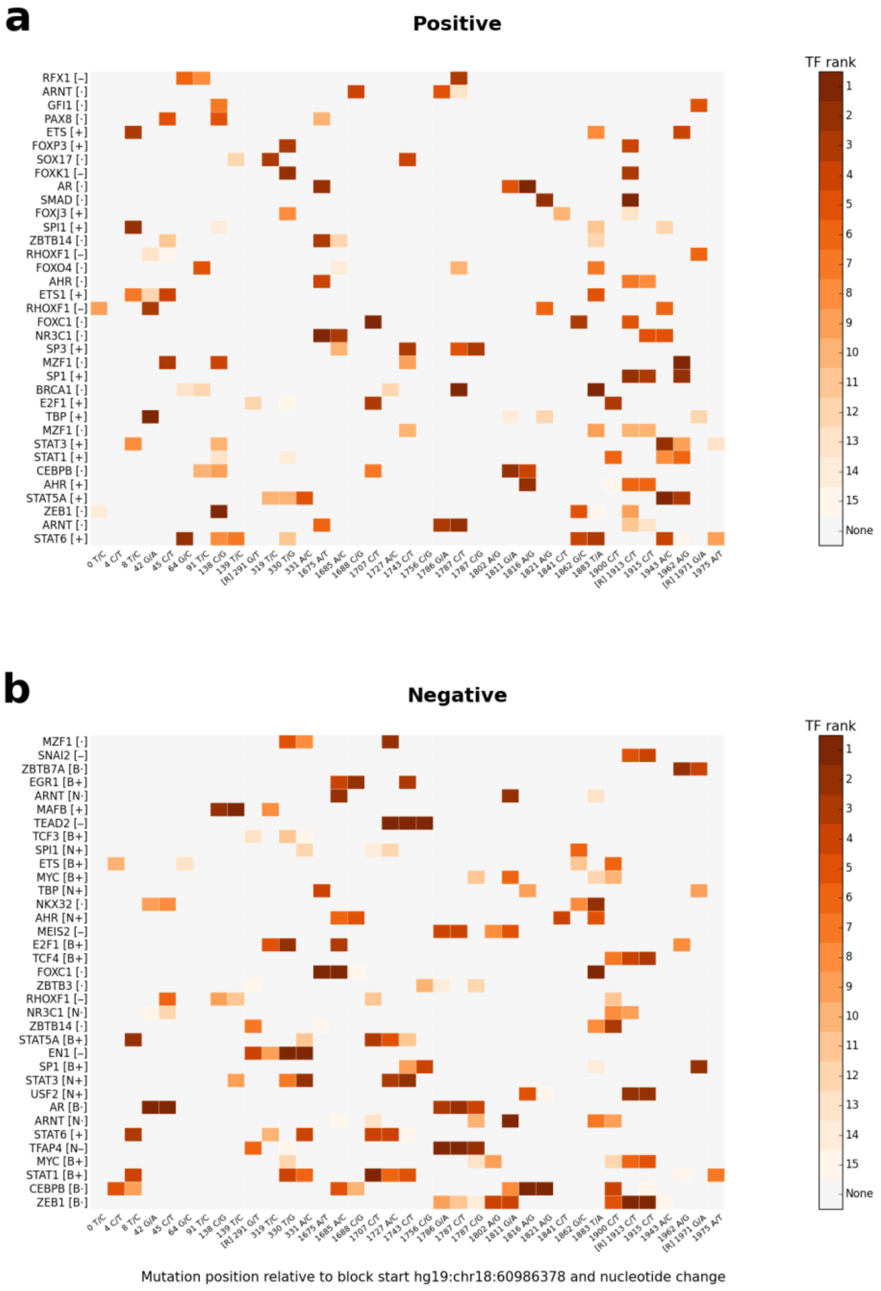

Supplementary Figure 5. TF affinity changes due to individual SNV at *BCL2* regulatory mutation block

a) Positive TF affinity changes due to individual SNV at *BCL2* regulatory mutation block; **b**) Negative TF affinity changes due to individual SNV at *BCL2* regulatory mutation block. Here, the row labels are the TF names, sorted by their frequency in FL patients. The color bar represents the rank order in the prediction: the lighter the color, the higher (less significant)

the ranking. In square brackets inside row labels: “+” means that the TF is highly expressed (top 25%), “•” means average expression, “-“ means low expression (lower 25%), “B” means that the TF has a peak in TSS ± 1kb region in the ReMap dataset, “N” means that the TF is in the ReMap dataset but has no peak nearby. [R] before mutation position means that this SNV is found in two patients. TFs with very low expression (RPKM < 0.03) were filtered out.

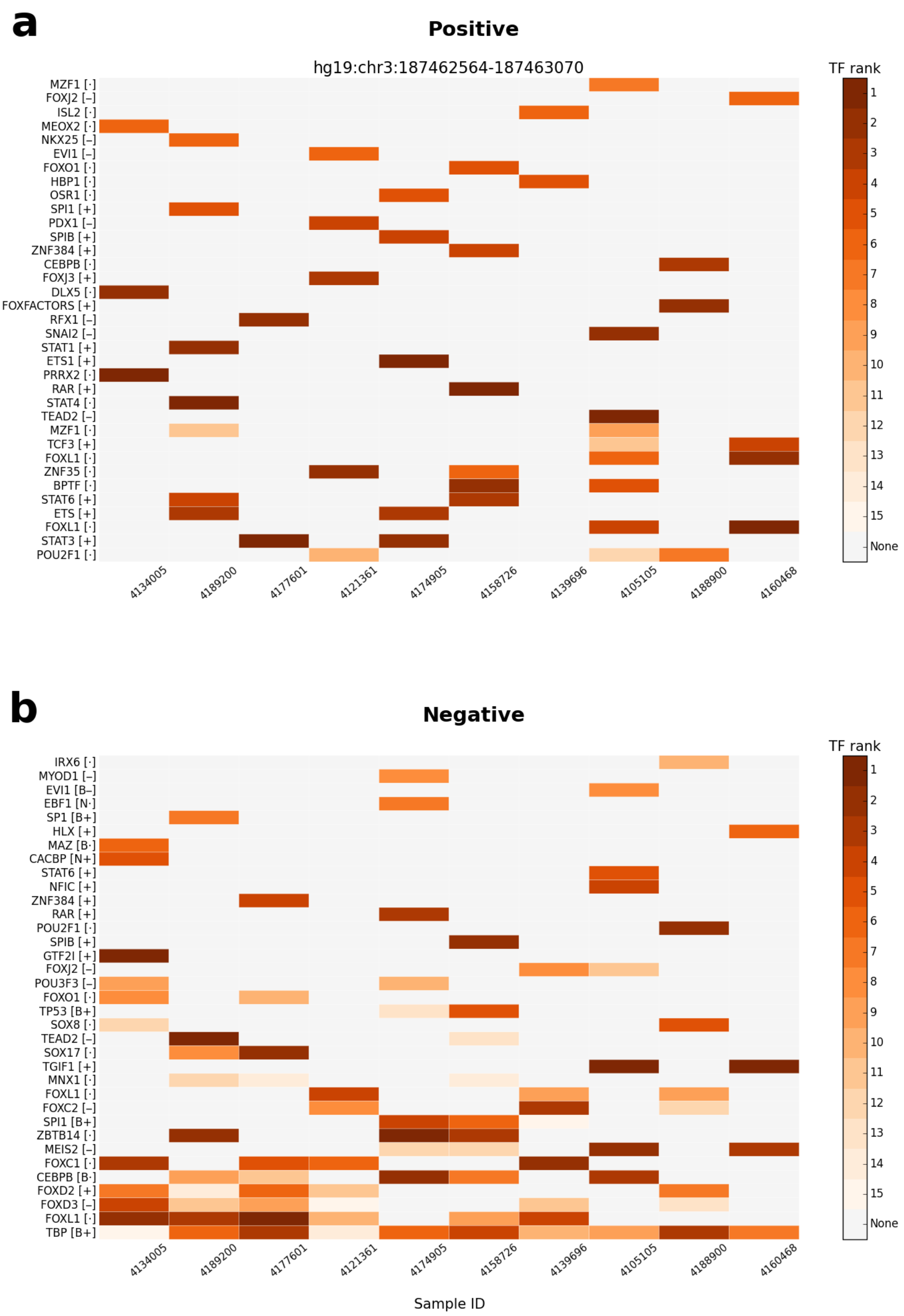

Supplementary Figure 6. TF affinity changes at *BCL6* patient-specific regulatory mutation block

a) Positive TF affinity changes at *BCL6* patient-specific regulatory mutation block; **b**) Negative TF affinity changes at *BCL6* patient-specific regulatory mutation block. Here, the row labels are the TF names, sorted by their frequency in 10 FL patients, and the column

labels are patients with the regulatory mutation block near *BCL6* TSS. The color bar represents the rank order in the prediction: the lighter the color, the higher (less significant) the ranking. In square brackets inside row labels: “+” means that the TF is highly expressed (top 25%), “•” means average expression, “-“ means low expression (lower 25%), “B” means that the TF has a peak in TSS ± 1kb region in the ReMap dataset, “N” means that the TF is in the ReMap dataset but has no peak nearby. TFs with very low expression (RPKM < 0.03) were filtered out.

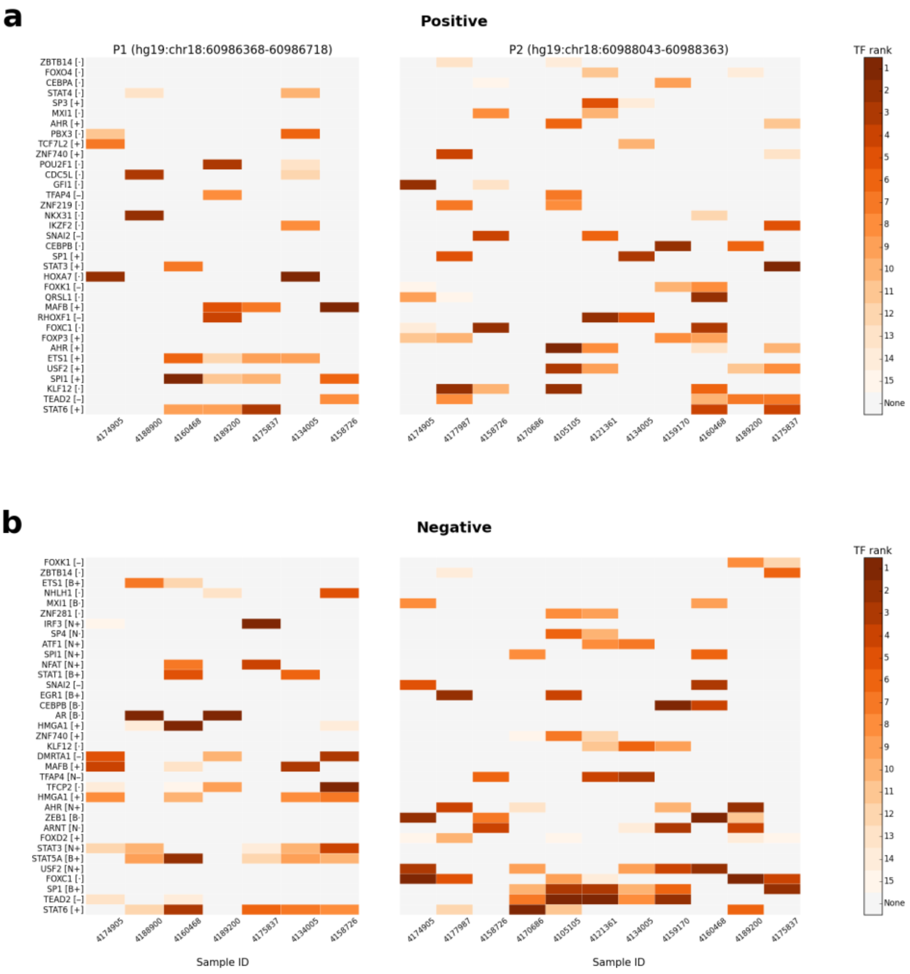

Supplementary Figure 7. TF affinity changes at *BCL2* patient-specific regulatory mutation blocks

a) Positive TF affinity changes at *BCL2* patient-specific regulatory mutation blocks; **b**) Negative TF affinity changes at *BCL2* patient-specific regulatory mutation blocks. Here, the row labels are the TF names, sorted by their frequency in FL patients, and the column labels are patients with the regulatory mutation block near *BCL2* TSS. The color bar represents the rank order in the prediction: the lighter the color, the higher (less significant) the ranking. In square brackets inside row labels: “+” means that the TF is highly expressed (top 25%), “•” means average expression, “-“ means low expression (lower 25%), “B” means that the TF has a peak in TSS ± 1kb region in the ReMap dataset, “N” means that the TF is in the ReMap

dataset but has no peak nearby. TFs with very low expression (RPKM < 0.03) were filtered out.

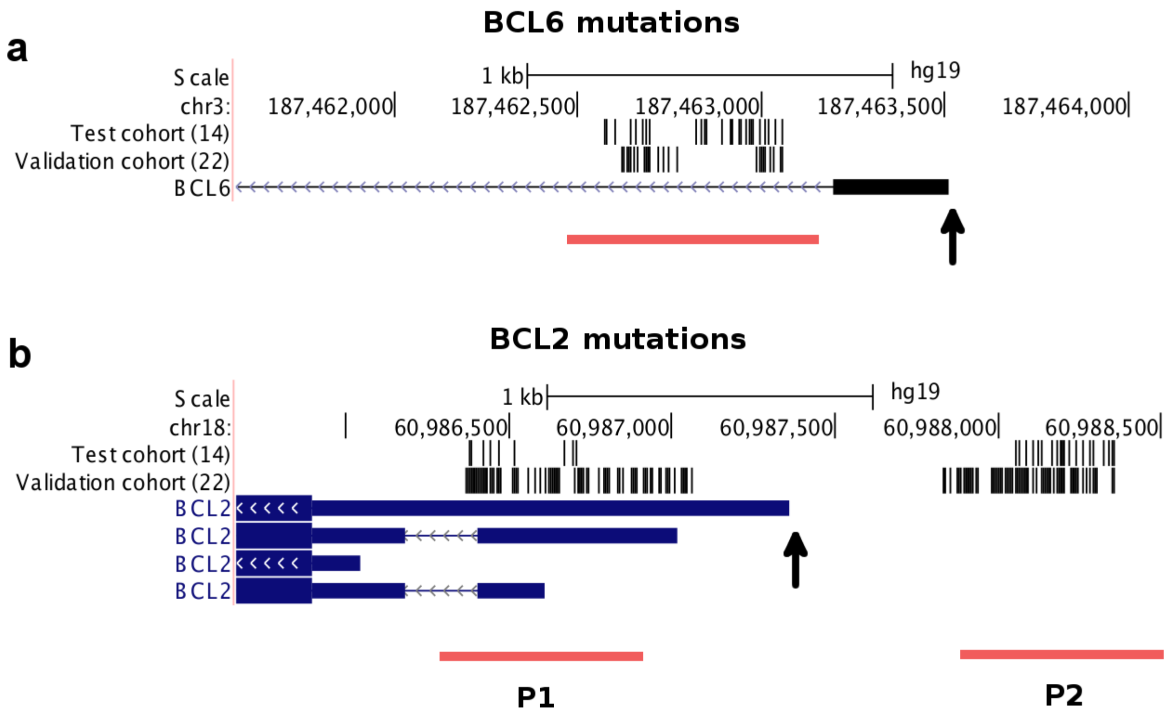

Supplementary Figure 8. Predicted *BCL6* and *BCL2* mutation blocks in both test and validation cohorts of FL patients.

a) Genome browser view of mutations within the *BCL6* mutation block detected by MuSSD in the test cohort of 14 patients and in the validation cohort of 22 patients. **b)** Genome browser view of mutations within the two *BCL2* mutation blocks detected in both cohorts. The reference genome annotations are displayed below, with solid bars representing exons and thin lines representing introns. Arrows on introns indicate direction of transcription. Black arrow marks the TSS. Red bars represent locations of the predicted regulatory

mutation blocks of *BCL6* and *BCL2*. **P1** and **P2** represent *BCL2* regulatory mutation blocks one and two, respectively.

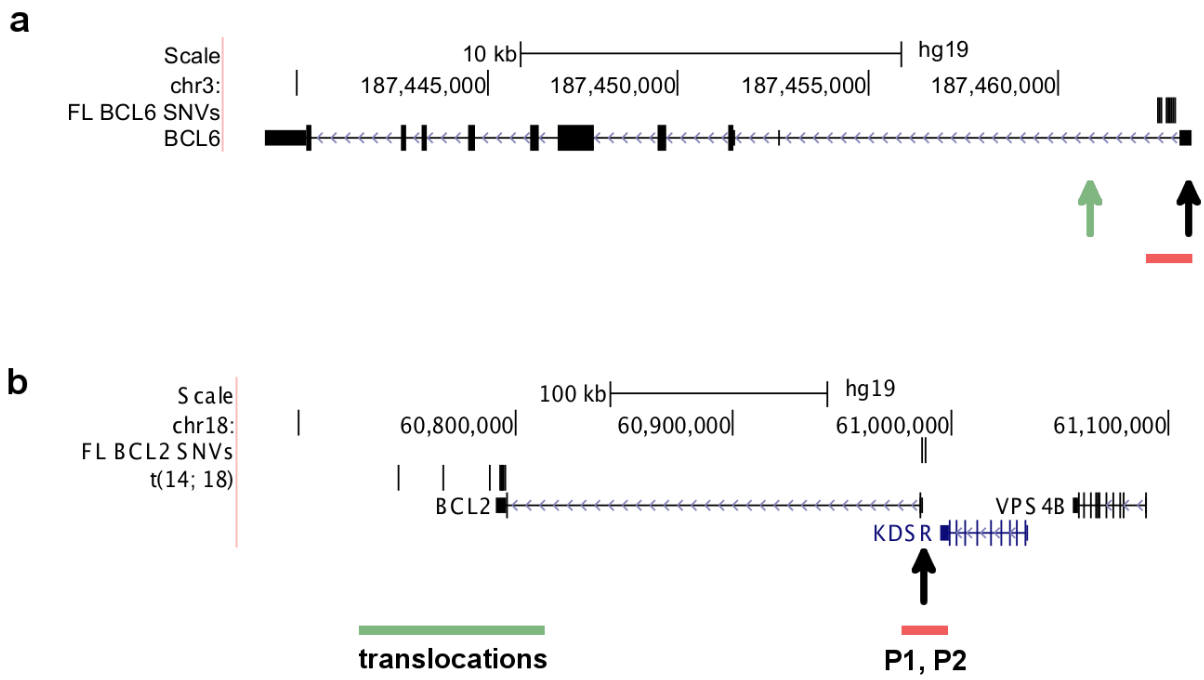

Supplementary Figure 9. Translocations in *BCL6* and *BCL2*.

a) Genome browser view of the detected *BCL6* translocation (green arrow). The regulatory mutation block of *BCL6* is marked by the red bar. The black arrow marks the *BCL6* TSS.

b) Genome browser overview of detected t(14; 18) breakpoints on chromosome 18. The two regulatory mutation blocks of *BCL2* are shown, as well as gene annotation. The black arrow marks *BCL2* TSS. Red and green bars represent the predicted regulatory mutation blocks and *IgH-BCL2* translocation sites, respectively.

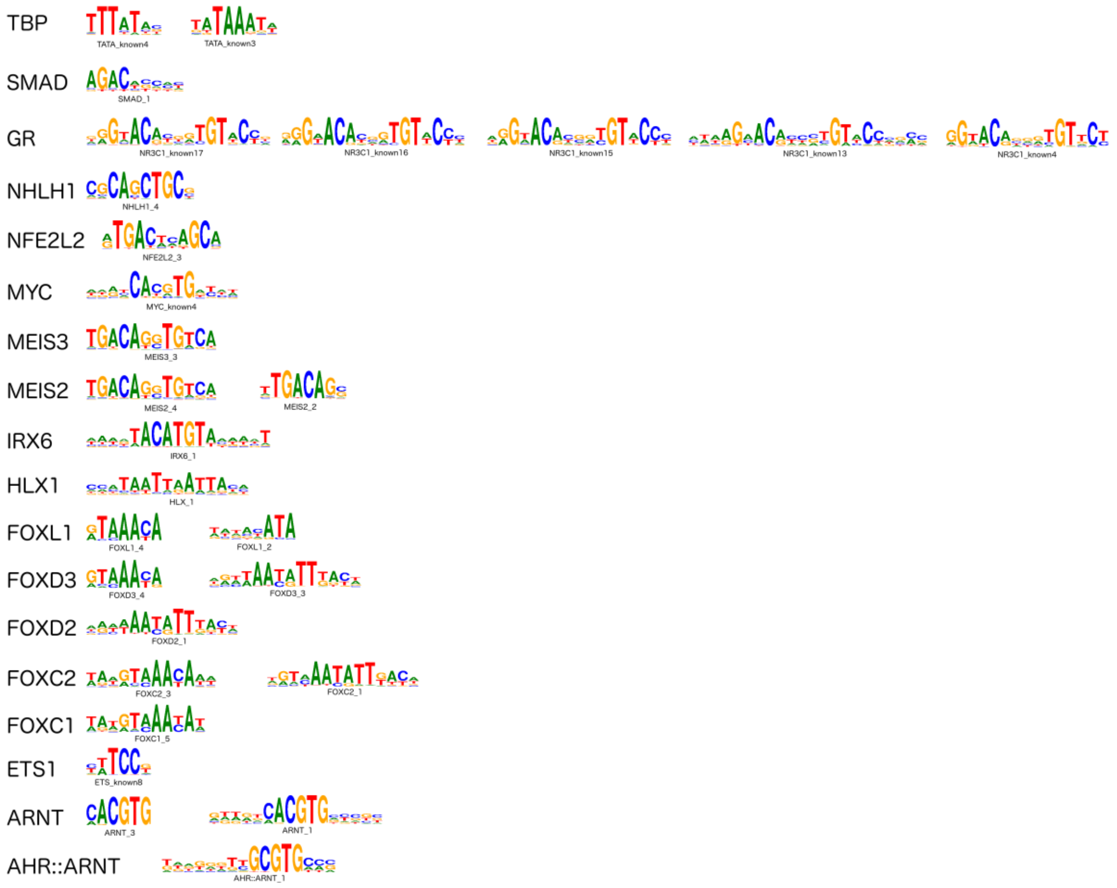

Supplementary Figure 10. PWM logos for significantly affected TFs.

For each PWM that is significantly affected in a regulatory mutation block, its logo is printed, grouped by TF name or family.
